# Surrogate modeling based on computational fluid dynamics for predicting the capsizing moment in a coupled shiplift system

**DOI:** 10.1371/journal.pone.0358034

**Published:** 2026-09-11

**Authors:** Tong Tang, Jianbao Yang, Zikang Hu, Junzhe You, Yang Zhang, Duanwei Shi

**Affiliations:** 1 Institute of Engineering and Technology, Hubei University of Science and Technology, Xianning, People's Republic of China; 2 College of International Business and Economics, Wuhan Textile University, Wuhan, China; Qingdao University, CHINA

## Abstract

Accurate prediction of the capsizing moment in shiplift systems remains a significant challenge, primarily due to the strong coupled interactions among the chamber, water body, and ship. Conventional three-dimensional (3D) numerical simulations are associated with high computational costs, while commonly used two-dimensional (2D) simplified models neglect ship effects, potentially leading to underestimation of the actual capsizing moment. In this study, a computational fluid dynamics (CFD)-informed surrogate model is developed for one-step-ahead prediction of capsizing moment in a coupled chamber-water-ship shiplift system. First, 3D models covering 19 scales are established, and the corresponding capsizing moments are obtained via CFD simulations. The CFD methodology is further validated against published smoothed particle hydrodynamics (SPH) results, with a maximum deviation of 4.1%, demonstrating the capability of the numerical framework to capture the relevant hydrodynamic responses. Furthermore, under El-Centro excitation, the peak capsizing moments predicted by the 3D model are substantially higher than those obtained using the conventional 2D simplified model for both the 3000 t light-load and 1350 t full-load conditions, indicating that 2D simplification may underestimate the capsizing moment in the examined cases and that three-dimensional effects should be considered when evaluating extreme responses. Based on the numerically generated CFD dataset, a hybrid surrogate framework is constructed, integrating convolutional neural networks, bidirectional long short-term memory networks, and random forests. To enhance the predictive robustness of the framework, multi-window isolation forest preprocessing, CNN-based feature enhancement, and parameter tuning based on the Mapping Mountain Gazelle Optimizer are employed. Comparisons with seven benchmark models demonstrate that the proposed model achieves the better overall predictive performance, with a mean absolute error of 0.0761 ± 0.0024, a root mean squared error of 0.1408 ± 0.0128, and a coefficient of determination of 0.9466 ± 0.0065 on the test set. Additional engineering cases indicate good generalization under ship-presence operating conditions, with peak prediction deviations below 4.8%. These results suggest that, when current and recent response states are available from monitoring or state-estimation systems, the proposed framework may support short-horizon capsizing-moment estimation.

## Introduction

**Fig 1:**
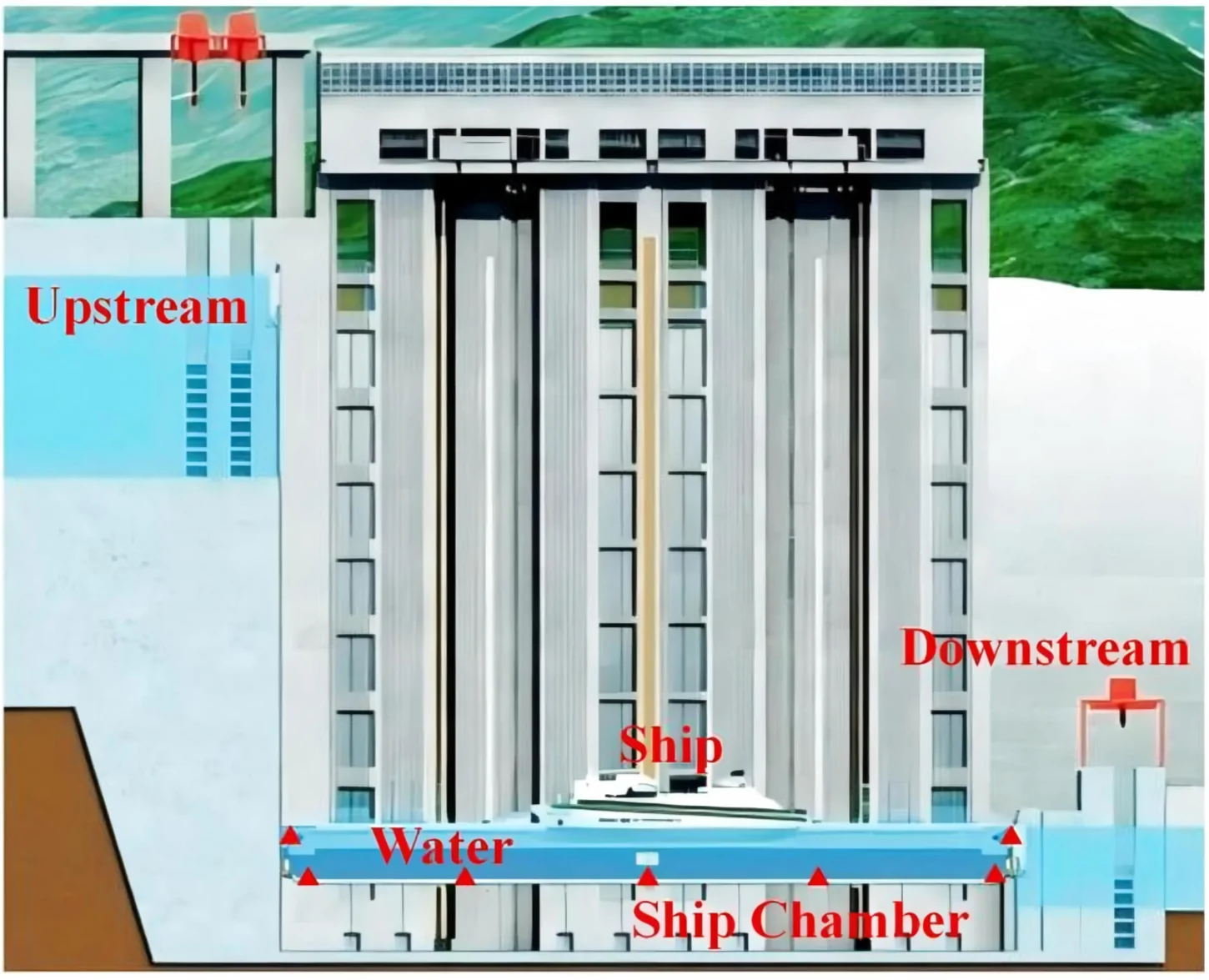
Schematic diagram of the shiplift structure.

Shiplifts are critical navigation facilities used to overcome water-level differences and to ensure safe and efficient ship passage in inland waterways, as illustrated in Fig 1. During operation, the chamber, water body, and ship interact as a strongly nonlinear coupled system, which makes accurate evaluation of the capsizing moment particularly challenging. High-fidelity three-dimensional (3D) numerical simulations can provide reliable predictions, but they are computationally expensive and therefore unsuitable for rapid engineering assessment. By contrast, most available analytical or empirical approaches are based on two-dimensional (2D) simplifications that neglect ship effects and may consequently underestimate the actual capsizing moment. An efficient and sufficiently accurate predictive framework is therefore needed to support rapid capsizing-moment assessment and short-horizon safety evaluation of chamber-water-ship coupled shiplift systems.

As a critical navigation infrastructure, the operational safety of a shiplift is fundamentally governed by the coupled dynamic behavior of the chamber-water-ship system within its ship chamber. Existing research has predominantly focused on engineering design and operational aspects. Several scholars have employed numerical simulations and theoretical analyses to investigate the draft subsidence law during ships entry and exit [1,2], the water-air-solid coupling behavior at the chamber bottom [3], and the associated hydrodynamic responses [4]. Other studies have examined the influence of structural parameter variations [5], longitudinal seismic excitations [6], and flood discharge conditions [7] on the dynamic characteristics of shiplifts, thereby providing a theoretical foundation for structural optimization and operational control [8]. In recent years, neural networks have demonstrated remarkable performance in marine and ocean engineering. Convolutional Neural Networks (CNNs) are now widely used in frequency domain analysis [9]. Meanwhile, Long Short-Term Memory (LSTM) networks, leveraging their superior temporal modeling capability, have proven highly effective in predicting ship whipping responses [10] and enabling millisecond-level real-time motion monitoring [11]. Building upon these advances, the development of CNN-LSTM (C-LSTM) spatiotemporal hybrid models has opened new avenues for predicting ship motions over short horizons under varying sea conditions [12,13]. Furthermore, attention mechanisms have enhanced models’ ability to focus on crucial information, thereby enabling real-time estimation of three-dimensional (3D) sloshing forces [14]. Building on this foundation, the CNN-Bidirectional Long Short-Term Memory (BiLSTM)-Attention (CNN-BiLSTM-Attention) model improves the efficiency of utilizing forward and backward temporal information, demonstrating superior performance in tasks such as ship heave prediction [15], analysis of tank pitch characteristics [16], and engine room ignition timing forecasting [17,18].In summary, although notable progress has been made in shiplift and ship motion research both domestically and internationally, existing studies remain largely confined to open-water ships or isolated physical scenarios. Theoretical investigations and real-time predictive methodologies specifically targeting the coupled chamber–water–ship system are still relatively underexplored.

While direct investigations into the coupled dynamic characteristics and predictive methodologies for the water–ship system within a shiplift chamber remain relatively scarce, a substantial body of research has accumulated in adjacent domains, particularly ship motion and sloshing dynamics in liquid containers. In the field of ship motion, researchers have employed Artificial Neural Networks (ANNs) for the rapid prediction of wave-induced ship loads [19] and ship speed [20]. By comparing various Recurrent Neural Network (RNN) architectures, approaches for predicting the torque of moving ships have been refined [21,22]. Systematic analyses of roll motion characteristics have further contributed to theoretical guidance for navigation safety [23]. Moreover, Physics-Informed Neural Networks (PINNs) [24,25] have demonstrated the ability to enhance predictive accuracy while preserving the underlying consistency of ship dynamics. Multilayer Perceptrons (MLPs) [26] and Multi-Fidelity Deep Neural Network (MFDNN) frameworks [27] have introduced novel perspectives for hydrodynamic performance evaluation in ship-form optimization. Cascaded CNNs have enabled end-to-end flow field analysis in water-entry experiments [28], thereby extending the applicability of data-driven methods. In the context of liquid sloshing in containers, both Residual Networks (ResNets) [29] and ANNs [30] have demonstrated strong performance in predicting sloshing loads. Hybrid approaches combining ANNs with traditional smoothed particle hydrodynamics (SPH) have yielded effective predictions of sloshing behavior in rectangular tanks [31,32]. Researchers have also applied ANN models grounded in finite element methods to estimate the dynamic response of water tanks [33] and the free-surface displacement in liquid tanks [34]. Notably, ANN-based models have achieved prediction errors within 10% for sloshing in oil–gas media [35], while Dendritic Artificial Neural Networks (D-ANNs) have proven effective for analyzing measurement data from LNG-fueled ships [36]. In addition, conventional methods such as Unsteady Reynolds-Averaged Navier–Stokes (URANS) simulations [37], potential-flow–computational fluid dynamics (CFD) coupling [38], SPH [39], and physical model experiments [40] continue to provide essential benchmarks for research on tank sloshing and ship motion. In summary, although a mature body of numerical methods and algorithmic frameworks has emerged in the fields of ship motion and liquid sloshing, the corresponding research objects differ substantially from the shiplift chamber system in terms of structural configuration, geometric scale, and operational conditions. Consequently, whether existing neural network architectures and training strategies are directly applicable to the dynamic analysis of shiplift systems remains an open question requiring further investigation.

To address these challenges, in this study, a CFD-informed surrogate model is developed for one-step-ahead prediction of capsizing moment in a chamber-water-ship coupled shiplift system. Nineteen in-service shiplifts with different scales are selected as representative cases. First, 3D chamber–water–ship models are established, and the corresponding capsizing moments are obtained through CFD simulations. The CFD methodology is further validated against published SPH simulations to evaluate its capability in reproducing the hydrodynamic response characteristics. A 19-case CFD database is subsequently constructed using consistent physical assumptions, boundary conditions, and solver settings, providing a unified numerical basis for the development and evaluation of the proposed surrogate model. Using this numerically generated CFD database, a hybrid surrogate model integrating a CNN, a BiLSTM, and a random forest (RF) is developed for efficient state-conditioned one-step-ahead prediction of capsizing moments. The CNN-BiLSTM branch is employed to extract nonlinear temporal and spatial feature dependencies from the constructed dynamic feature representations, whereas the RF branch provides complementary nonlinear regression capability through ensemble learning. The predictive performance, robustness, and applicability of the proposed framework are systematically evaluated through benchmark comparisons, ablation studies, and independent engineering-case validations. When current and recent system states are available from monitoring or state-estimation systems, the developed surrogate model can provide rapid short-horizon capsizing-moment estimates, offering a computationally efficient tool for operational assessment of chamber-water-ship coupled shiplift systems.

## Models and methods

### Physical model

**Fig 2:**
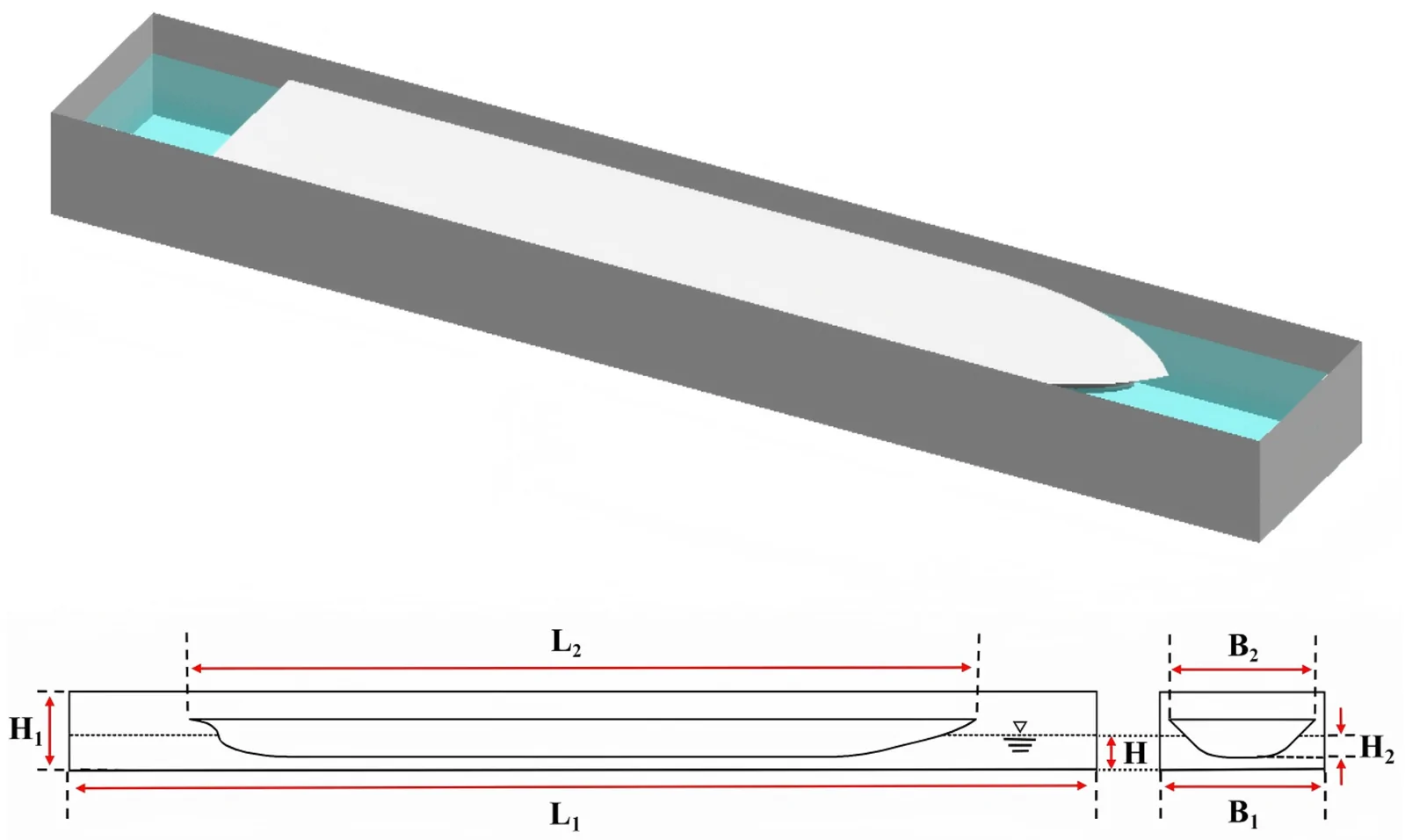
Axonometric and 2D cross-sectional views of the shiplift.

During shiplift operations, the most adverse condition occurs when the ship chamber is situated at the uppermost section of the tower column in a docked and locked state, especially under seismic excitation [41]. Under such conditions, the tower column amplifies the seismic excitation and transmits it to the ship chamber via the guiding mechanism, thereby influencing the dynamic characteristics of the water body and the ship inside the chamber. In light of this, this study takes nineteen in-service shiplifts worldwide as the research objects (see Table 1), to investigate the capsizing moment characteristics of the chamber-water-ship system under seismic excitation, considering different sizes and ship tonnages. The ship drafts listed in Table 1 do not necessarily represent the maximum-capacity full-load condition for all shiplifts. Full-load ship parameters were adopted where such information was explicitly available in the original engineering sources; otherwise, the reported design or representative operating drafts were used. For physical model construction, the ship chamber was simplified as a rectangular shape [1], and the ship was equivalently represented based on typical cargo ships [42]. Fig 2. illustrates the overall model, where L_1_, B_1_, and H_1_ respectively represent the length, width, and height of the ship chamber, and H represents the liquid level height; L_2_, B_2_, and H_2_ respectively represent the length, width, and draft of the ship.

**Table 1:** Statistics of nineteen large and medium-sized in-service shiplifts worldwide.

No.	Name	Country	Head (m)	Capacity (t)	Chamber dimensions L_1_ × B_1_ × H_1_ (m)	Ship principal dimensions L_2_ × B_2_ × H_2_ (m)
1	Krasnoyarsk	Russia	101	2000	90 × 18 × 3.3	86 × 13.5 × 4.9
2	Sterrebeek	Belgium	73.0	1350	112 × 12 × 3.35	110 × 11.4 × 2.8
3	Lokeren	Belgium	67.7	1350	87 × 12 × 3	85 × 9.5 × 2.5
4	Lüneburg	Germany	38.0	1350	100 × 12 × 3.5	85 × 9.5 × 2.5
5	Hindenburg	Germany	14.5	1350	90 × 12 × 3	85 × 9.5 × 2.5
6	Niederfinow	Germany	36.0	1000	85 × 12 × 2.5	68 × 9.5 × 1.9
7	Rostock	Germany	18.6	1000	85 × 12 × 2.5	68 × 9.5 × 1.9
8	Three Gorges	China	113	3000	120 × 18 × 3.5	84.5 × 16.2 × 2.65
9	Shuikou	China	59.0	2500	114 × 12 × 2.5	45 × 10.8 × 1.6
10	Baise	China	88.8	1000	130 × 12 × 3.9	68 × 11 × 2.4
11	Longtan	China	179	1000	73 × 12.2 × 3.5	68 × 11 × 2.4
12	Tingzikou	China	85.4	1000	116 × 12 × 2.5	45 × 10 × 1.6
13	Siling	China	76.7	500	58 × 12 × 2.5	55 × 10.8 × 1.6
14	Pengshui	China	66.6	500	59 × 11.7 × 2.5	55 × 10.8 × 1.6
15	Shatuo	China	75.4	500	64 × 12 × 2.5	55 × 10.8 × 1.6
16	Goupitan	China	130	500	59 × 12 × 2.3	55.8 × 10.8 × 1.6
17	Gaobazhou	China	40.3	300	42 × 10.2 × 1.7	35 × 9.2 × 1.3
18	Geheyuan	China	122	300	42 × 10.2 × 1.7	35 × 9.2 × 1.3
19	Danjiangkou	China	62.0	300	42 × 10.2 × 1.7	40 × 9.2 × 1.4

### Mathematical model

To investigate the sloshing characteristics of the chamber-water-ship system, 3D numerical simulations were conducted using Ansys Fluent 2022 R1. Among these, the continuity equation takes the following form [43]:


$$\begin{array}{c} \nabla \cdot u=\text{0} \end{array}$$ (1)

where u=(*u*, *v*, *w*) is the velocity vector, with *u*, *v*, *w* representing its components along the *x*, *y*, *z* spatial coordinates,respectively. The Navier-Stokes equations are presented as follows [44]:


$$\begin{array}{c} \frac{\partial u}{\partial t}+(u\cdot \nabla )u=-\frac{\text{1}}{\rho }\nabla p+\nu \nabla \text{2}u+\frac{f}{\rho } \end{array}$$ (2)

where *p* is the pressure field, *ρ* is the fluid density, *t* is time, *v* is the kinematic viscosity, andf=(*fx*, *fy*, *fz*) is the body force vector, with *fx*, *fy*, *fz* representing the external force components along the *x*, *y*, *z* directions, respectively.

The equations of motion for a ship are as follows [45]. For translation motion:


$$\begin{array}{c} m\frac{{\text{d}}^{\text{2}}r}{\text{d}t^{\text{2}}}=F \end{array}$$ (3)

where *m* is the ship mass, r=(*r*_*x*_, *r*_*y*_, *r*_*z*_) is the position vector of the center of mass, with *r*_*x*_, *r*_*y*_, *r*_*z*_ denoting its components along the *x*, *y*, *z* axes; F=(*F*_*x*_, *F*_*y*_, *F*_*z*_) is the total external force vector, with *F*_*x*_, *F*_*y*_, *F*_*z*_ represent the force components acting on the ship in the *x*, *y*, *z* directions. For rotation motion:


$$\begin{array}{c} I\frac{\text{d}\omega }{\text{d}t}+\omega \times (I\omega )=M \end{array}$$ (4)

where I is the moment of inertia tensor, ω=(*ω*_*x*_, *ω*_*y*_, *ω*_*z*_) is the angular velocity vector, with *ω*_*x*_, *ω*_*y*_, *ω*_*z*_ corresponding to the angular velocity components about the *x*, *y*, *z* axes; M=(*M*_*x*_, *M*_*y*_, *M*_*z*_) denotes the total external torque vector, where *M*_*x*_, *M*_*y*_, *M*_*z*_ are the external torque components acting on the ship about the *x*, *y*, *z* axes.

### Simulation settings for coupled motion of chamber-water-ship

**Fig 3:**
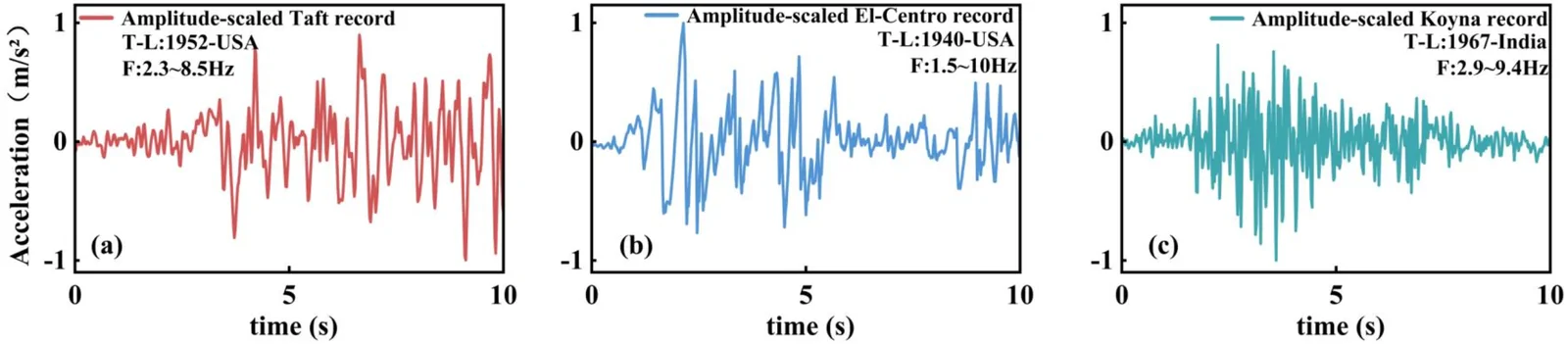
Acceleration time histories of the seismic records considered in this study: (a) Taft and (c) Koyna, used only for external validation, and (b) El-Centro, used to generate the 19-case model-development dataset.

This study employs a transient solver to simulate the coupled motion of the chamber-water-ship system. The default environment is set to atmospheric pressure, and the dynamic viscosity of water is specified as 0.001 kg/(m·s). Time discretization relies on an implicit scheme, complemented by implicit body force updating to enhance convergence and stability. A time step of 0.001 s is applied, with an iterative residual convergence criterion of 10^−5^ [46]. Considering the transient characteristics of the fluid, the SST k − ω model is employed to characterize the turbulent behavior of the fluid [47], and the Volume of Fluid (VOF) method [48] is utilized to track the gas-liquid interface evolution. The computational domain uses a circular-polygonal overlapping grid structure combined with dynamic grid technology to accurately handle moving boundaries variations. Both the ship and the ship chamber are modeled as rigid bodies, with the heat exchange effect neglected. The 19-case CFD database used for model development was generated exclusively under the El-Centro seismic record scaled to a PGA of 0.1g. The Taft and Koyna records shown in Fig 3 were not included in model training, validation, or internal testing; they were reserved for external engineering-case validation. In the external validation, the Taft and Koyna records were scaled to PGAs of 0.05g and 0.2g, respectively. For consistency with practical engineering requirements, the more hazardous 0 ~ 10s data segment is selected for calculations. The ship’s motion is defined via a User-Defined Function (UDF), and the fluid loads on its surface are transmitted in real time to a six-degree-of-freedom solver to achieve the positioning of rigid body motion. To prevent grid distortion, rezoning and smoothing are applied to the water-ship interface region. Under initial conditions, the ship rests statically in the water body, and neither has any initial velocity.

### Numerical accuracy verification

#### Grid independence analysis.

**Fig 4:**
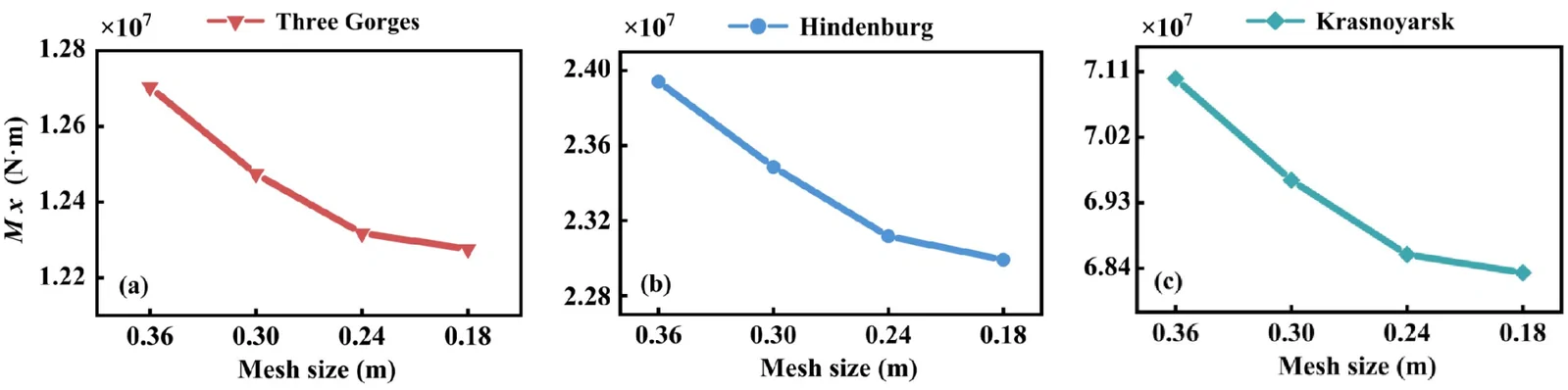
Grid independence analysis based on the averaged peak longitudinal capsizing moments for three representative shiplift cases. (a) Three Gorges, (b) Hindenburg, and (c) Krasnoyarsk.

Taking the ship chambers of the Three Gorges, Hindenburg, and Krasnoyarsk shiplifts as examples, El-Centro seismic waves were applied to each chamber separately, ensuring identical excitation magnitude and direction. Subsequently, the four largest positive local maxima of the longitudinal capsizing moment *M*_*x*_ at the center of the shiplift chamber bottom were extracted from the CFD-generated time histories. These peak values were ranked according to their magnitudes, corresponding to the largest, second-largest, third-largest, and fourth-largest positive local maxima, and their average value was adopted as the comparison indicator. The results are shown in Fig 4.

As illustrated in Fig 4, with grid refinement, the discrepancies among the calculated average values of the selected longitudinal capsizing-moment peaks gradually decrease and tend to stabilize. In particular, when the grid size decreases from 0.24m to 0.18m, the relative errors of the averaged peak values for all three cases remain below 1%. It should be noted that 0.24m represents the maximum grid size adopted among the 19 models rather than a fixed grid size uniformly applied to all cases. For smaller shiplift chambers, finer grids (down to 0.1m) were employed to ensure sufficient spatial resolution. The total number of grid cells for all 19 models was maintained within approximately 1.1–1.5 million. Therefore, considering both computational efficiency and numerical accuracy, a maximum grid size of 0.24m was determined as the upper limit of mesh resolution for subsequent simulations.

#### Time step independence analysis.

**Fig 5:**
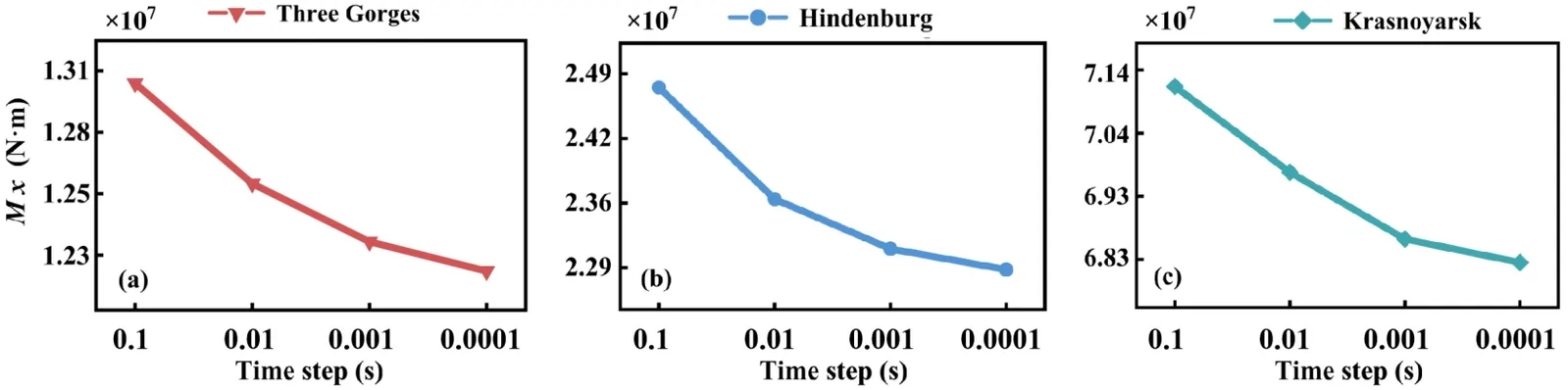
Time-step independence analysis based on the averaged peak longitudinal capsizing moments for three representative shiplift cases. (a) Three Gorges, (b) Hindenburg, and (c) Krasnoyarsk.

To investigate whether the selected temporal resolution is sufficient to accurately capture the transient characteristics of the capsizing moment, a time-step sensitivity analysis was performed. The Three Gorges, Hindenburg, and Krasnoyarsk shiplifts were selected as representative cases, and identical El-Centro seismic excitation was applied under the same boundary conditions. Four time steps, namely 0.1s, 0.01s, 0.001s, and 0.0001s, were examined. For each case, the longitudinal capsizing moment *M*_*x*_ at the center of the chamber bottom was extracted from the transient CFD simulations, and the average value of the four largest positive local maxima was adopted as the characteristic indicator. The comparison results are presented in Fig 5.

As shown in Fig 5, relatively large time steps result in noticeable variations in the calculated peak capsizing moments, indicating that insufficient temporal resolution may affect the accuracy of transient load evaluation. With further refinement of the time step, the calculated peak values gradually converge. When the time step was reduced from 0.001s to 0.0001s, the relative differences in the averaged peak moments for all three cases remained below 2%, indicating that further temporal refinement produced negligible changes. Therefore, a time step of 0.001s was selected for subsequent CFD simulations considering both transient response accuracy and computational efficiency.

### Algorithm theoretical basis

In this study, the input feature vector *X*_t_ consists of ten physically meaningful features. Specifically, the input at prediction instant t is defined as: *X*_t_=[L_2_/L_1_, B_2_/B_1_, H_2_/H, H/H_1_, *a*_(t-1)_, *a*_(t)_, *F*_(t-1)_, *F*_(t)_, *M*_(t-1)_, *M*_(t)_]. The first four components represent the geometric characteristics of the ship chamber configuration, including the ratios of ship length to chamber length (L_2_/L_1_), ship width to chamber width (B_2_/B_1_), ship draft to liquid height (H_2_/H), and liquid height to chamber height (H/H_1_). The remaining six components describe the available historical dynamic responses, including acceleration (*a*_(t-1)_, *a*_(t)_), capsizing force (*F*_(t-1)_, *F*_(t)_), and capsizing moment (*M*_(t-1)_, *M*_(t)_) at the current and previous sampling instants. The prediction target is the capsizing moment at the next sampling instant *M*_(t+1)_. Therefore, the proposed model performs one-step-ahead prediction, where only the information available at or before the current instant t is used to estimate the response at t + 1. The prediction horizon corresponds to one sampling interval ∆t, rather than a long-term multi-step forecasting process. In the machine-learning dataset, the sampling interval is ∆t = 0.02 s; therefore, *M*_(t+1)_ represents the capsizing moment 0.02 s after the current prediction instant. During practical applications, the prediction process can be continuously updated as new measurements become available, enabling short-horizon capsizing moment monitoring. Although the input vector contains dynamic responses from two adjacent sampling instants (t − 1 and t), it is organized as an ordered feature representation rather than a long temporal sequence. Therefore, the CNN module focuses on extracting nonlinear correlations among physically related variables within the feature arrangement, while the BiLSTM module further captures the dependency information embedded in the extracted feature representations.

For the convolution operation, *i* represents the position of the convolution window in the input feature dimension and *j* represents the position of the feature within the convolution kernel. Therefore, *X*_1_, *X*_2_,..., *X*_10_ correspond to the ten input features listed above. The convolution kernel moves along the feature dimension to extract local nonlinear interactions within the organized feature representation. For example, when the kernel covers a subset of feature representations, the convolution operation aggregates their combined information to identify potential interactions among geometric and dynamic response variables.

#### Convolutional neural network.

The CNN adopts “convolution (Conv) - batch normalization (BatchNorm) - activation function (ReLU) - max pooling (MaxPool)” as its basic unit to extract local nonlinear interactions from the input feature vector. The one-dimensional convolution operation is applied along the feature dimension. For the input vector *X*_t_=[*X*_1_, *X*_2_,..., *X*_*L*_], where *L* = 10 and each element corresponds to one physically meaningful variable defined above, the one-dimensional convolution operation is expressed as [49]:


$$\begin{array}{c} C_{i}=f\left(\sum_{j=\text{1}}^{\text{h}}W_{j}\cdot X_{i+j-\text{1}}+b\right) \end{array}$$ (5)

where $C_{i}$ denotes the *i*-th element of the output feature map, $W_{j}$ represents the weight at the *j*-th position of the convolution kernel, and $X_{i+j-\text{1}}$ denotes the (*i* + *j* − 1)-th element of the input feature vector. In this study, *X*_1_-*X*_10_ correspond to the ten physically meaningful variables describing the chamber-water-ship coupled system. Here, *i* represents the starting position of the convolution window, while *j* indicates the relative position within the kernel. *h* represents the size of the convolution kernel, which is set to 2 in this study, *b* is the bias term and f(·) is the activation function.

The extracted feature set after convolution is expressed as:


$$\begin{array}{c} C=\{C_{\text{1}},C_{\text{2}},\ldots ,C_{L-h+\text{1}}\} \end{array}$$ (6)

where *L* represents the length of the input feature vector, In this study, the kernel size is set to *h* = 2, resulting in an output feature length of *L* − *h* + 1 = 9. Each convolution operation combines two feature elements within the organized representation to construct higher-level nonlinear feature representations. These representations characterize potential coupled relationships among geometric characteristics and dynamic response variables of the chamber-water-ship system.

The max-pooling operation further compresses the feature dimension by retaining dominant feature responses:


$$\begin{array}{c} C_{pool}=\text{max}\{C_{\text{1}},C_{\text{2}},\ldots ,C_{m}\} \end{array}$$ (7)

where $C_{pool}$ denotes the output feature vector after pooling and *m* represents the size of the pooling window, which is set to 2 in this study. Through these operations, CNN extracts compact and informative feature representations from the original input variables, which are subsequently fed into the BiLSTM module for further feature encoding.

#### BiLSTM neural network.

The BiLSTM module is employed to further encode the feature representations extracted by CNN and capture the internal dependencies among these representations. In this study, BiLSTM does not directly process future physical responses; instead, it operates on the feature representation generated from the observed information at and before the prediction instant t. Therefore, the backward direction of BiLSTM operates only within the extracted feature representation space and does not introduce unavailable future measurements.

The LSTM unit updates the hidden state through three gating mechanisms, including the forget gate, input gate, and output gate. The forget gate determines which information from the previous hidden state should be retained [50]:


$$\begin{array}{c} f_{t}=\sigma (W_{f}\cdot [h_{t-\text{1}};x_{t}]+b_{f}) \end{array}$$ (8)

where *x*_*t*_ represents the CNN-extracted feature representation at the corresponding feature position, *h*_*t*−1_ denotes the previous hidden state, *σ*(·) represents the sigmoid activation function; *f*_*t*_, *W*_*f*_, and *b*_*f*_ are the output vector, weight matrix, and bias vector of the forget gate, respectively.

Then, the input gate controls the incorporation of new information and updates the cell state:


$$\begin{array}{c} i_{t}=\sigma (W_{i}\cdot [h_{t-\text{1}},x_{t}]+b_{i}) \end{array}$$ (9)

$$\begin{array}{c} {\tilde{C}}_{t}=\text{tanh}(W_{C}\cdot [h_{t-\text{1}},x_{t}]+b_{C}) \end{array}$$ (10)

$$\begin{array}{c} C_{t}=f_{t}⊙C_{t-\text{1}}+i_{t}⊙{\tilde{C}}_{t} \end{array}$$ (11)

where $i_{t}$, $W_{i}$, and $b_{i}$ are the output vector, weight matrix, and bias vector of the input gate, respectively; ${\tilde{C}}_{t}$, $W_{C}$, and $b_{C}$ denote the candidate cell state vector and its associated weight matrix, and bias vector; *C*_*t*_ represents the updated cell state vector of the LSTM unit, where “cell” refers to the neural network memory unit rather than the computational cells in the CFD mesh, and ⊙ represents the element-wise multiplication.

Subsequently, the output gate controls the information transferred from the cell state to the hidden state:


$$\begin{array}{c} o_{t}=\sigma (W_{o}\cdot [h_{t-\text{1}},x_{t}]+b_{o}) \end{array}$$ (12)

$$\begin{array}{c} h_{t}=o_{t}⊙\text{tanh}(C_{t}) \end{array}$$ (13)

where $o_{t}$, $W_{o}$, and $b_{o}$ are the output vector, weight matrix, and bias vector of the output gate, respectively.The hidden state $h_{t}$ represents the encoded high-level dynamic representation used for subsequent prediction.

Finally, to enhance feature representation capability, BiLSTM adopts two parallel LSTM layers with opposite processing directions. The forward and backward hidden states are concatenated:


$$\begin{array}{c} H_{t}=[{\vec{h}}_{t};{\overset{\leftarrow }{h}}_{t}] \end{array}$$ (14)

where ${\vec{h}}_{t}$ and ${\overset{\leftarrow }{h}}_{t}$ denote the hidden states generated by the forward and backward LSTM layers, respectively. The concatenated vector $H_{t}$ provides a comprehensive representation of the nonlinear dependencies within the ordered CNN-extracted feature representations and is subsequently transmitted to the regression module for capsizing moment prediction. [·] represents the vector concatenation operation.

#### Random forest.

Unlike the CNN-BiLSTM branch, which extracts nonlinear feature representations from the input variables, the RF branch is constructed as an independent regression module to provide an additional prediction result. The RF and CNN-BiLSTM branches operate in parallel, and their outputs are subsequently integrated through the fusion layer to obtain the final prediction of the capsizing moment *M*_(t+1)_. RF includes three core processes: “Bootstrap Sampling - Independent Tree Regression - Prediction Aggregation”. The input dataset is defined as [51]:


$$\begin{array}{c} D=\{(x_{\text{1}},y_{\text{1}}),(x_{\text{2}},y_{\text{2}}),...,(x_{N},y_{N})\} \end{array}$$ (15)

where $x_{N}$ denotes the input feature vector of the *N*-th sample, and $y_{N}$ represents the corresponding target value, namely the capsizing moment *M*_*N*(t+1_) at the next prediction instant. Each feature vector contains ten input variables, including four geometric ratios and six historical dynamic responses.

The bootstrap sampling process generates *K* different training subsets from the original dataset through sampling with replacement:


$$\begin{array}{c} D_{k}=Bootstrap(D),k=\text{1},\text{2},...,K \end{array}$$ (16)

where $D_{k}$ represents the training subset used for constructing the *k*-th regression tree.

Subsequently, each subset is independently used to train a regression tree:


$$\begin{array}{c} T_{k}:x\mapsto {\hat{y}}_{k}(x) \end{array}$$ (17)

where $T_{k}$ denotes the *k*-th regression tree, and ${\hat{y}}_{k}(x)$ represents the prediction output of the corresponding tree for input vector *x*. Each regression tree establishes a nonlinear mapping between the input variables and the target capsizing moment, and ↦ denotes the mapping symbol.

Finally, to improve prediction robustness and reduce the influence of individual trees, the outputs from all regression trees are aggregated by averaging:


$$\begin{array}{c} \hat{y}(x)=\frac{\text{1}}{K}\sum_{k=\text{1}}^{k}{\hat{y}}_{k}(x) \end{array}$$ (18)

where $\hat{y}(x)$ denotes the final prediction generated by the RF branch.

### C-BL-R neural network model

**Fig 6:**
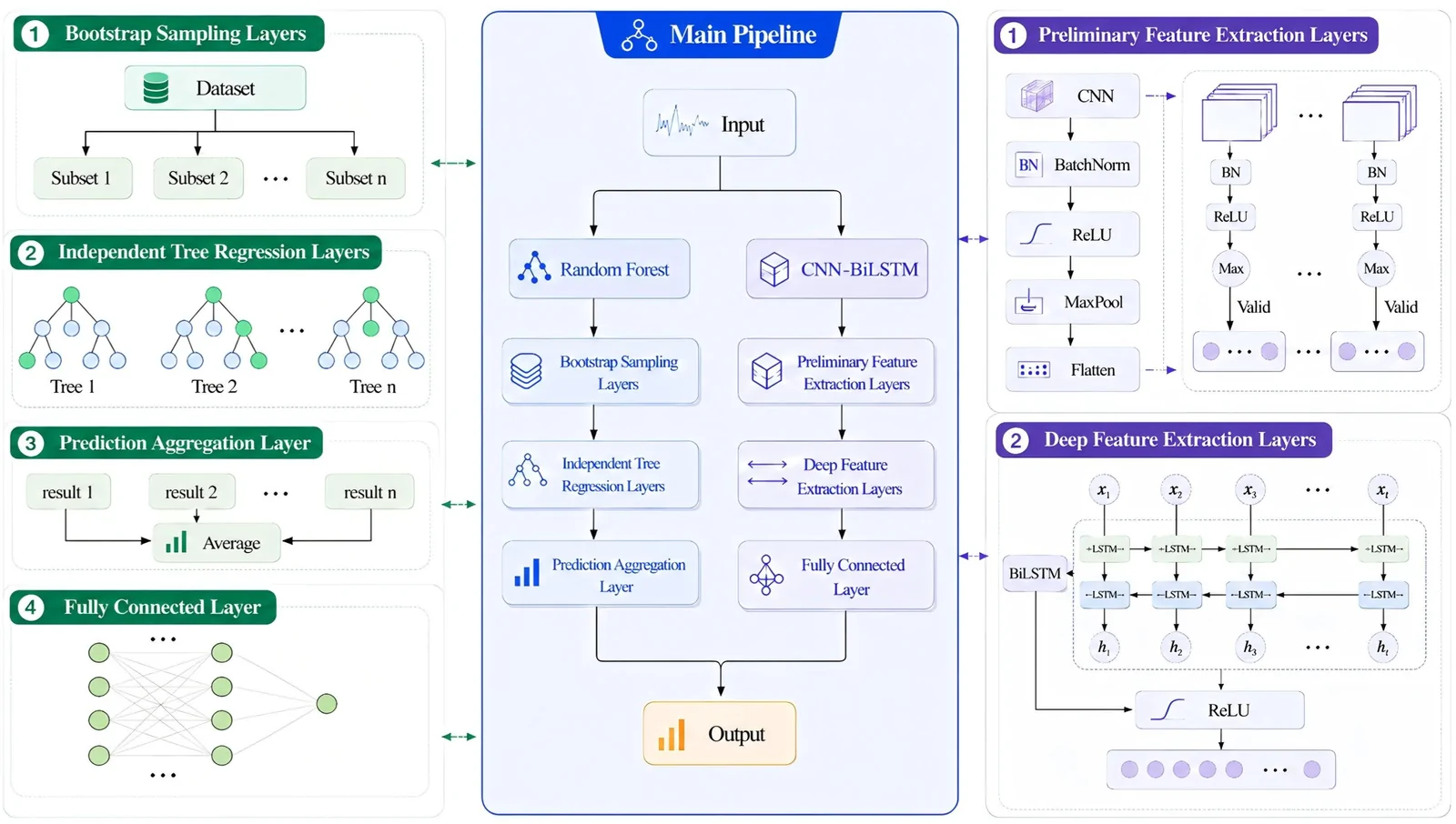
Schematic diagram of the C-BL-R hybrid model framework.

This study proposes a hybrid Convolutional Neural Network-Bidirectional Long Short Term Memory-Random Forest (C-BL-R) surrogate model for one-step-ahead capsizing moment prediction in the chamber-water-ship coupled shiplift system, as illustrated in Fig 6. The proposed framework consists of two parallel regression branches: a CNN-BiLSTM (C-BL) branch and an independent RF branch. Prior to model training, eight data-driven features are generated from the 10 original physical variables through an auxiliary CNN-based feature-enhancement procedure and concatenated with the original variables. Consequently, both the C-BL and RF branches receive the same 18-dimensional input representation. The outputs of the two branches are subsequently combined through an adaptive weighted fusion strategy to obtain the final prediction of the capsizing moment *M*(t + 1).

The two branches are designed to provide complementary predictions through heterogeneous learning mechanisms. The C-BL branch extracts nonlinear dependencies from the structured input representation through deep feature learning, whereas the RF branch provides an independent ensemble-regression prediction that is relatively robust to sample fluctuations. Output-level weighted fusion was selected instead of feature-level concatenation because the two branches do not naturally generate homogeneous intermediate representations. Direct concatenation would therefore require additional feature-alignment and regression layers, increasing architectural complexity and reducing the independence of the two predictors. A stacking-type ensemble would require an additional meta-learner trained using out-of-fold predictions from the base models, thereby introducing another training stage and additional parameters. In contrast, the adopted weighted fusion directly combines the two scalar capsizing-moment predictions, preserves the independence of the two branches, and provides an explicit and interpretable representation of their relative contributions.

The fusion weights are designed as learnable network parameters, which are synchronously optimized with the backbone network parameters through backpropagation during the training process. The initial weights are set to 0.5, and the optimal fusion weights determined by adaptive optimization are approximately ωC-BL = 0.49 and ωRF = 0.51. This mechanism enables the model to dynamically adjust the contribution ratio of the two branches according to the optimization objective during training, allowing the fusion layer to automatically learn the optimal balance between the two complementary predictors, achieving complementary correction of bias and improving overall prediction accuracy.

**Fig 7:**
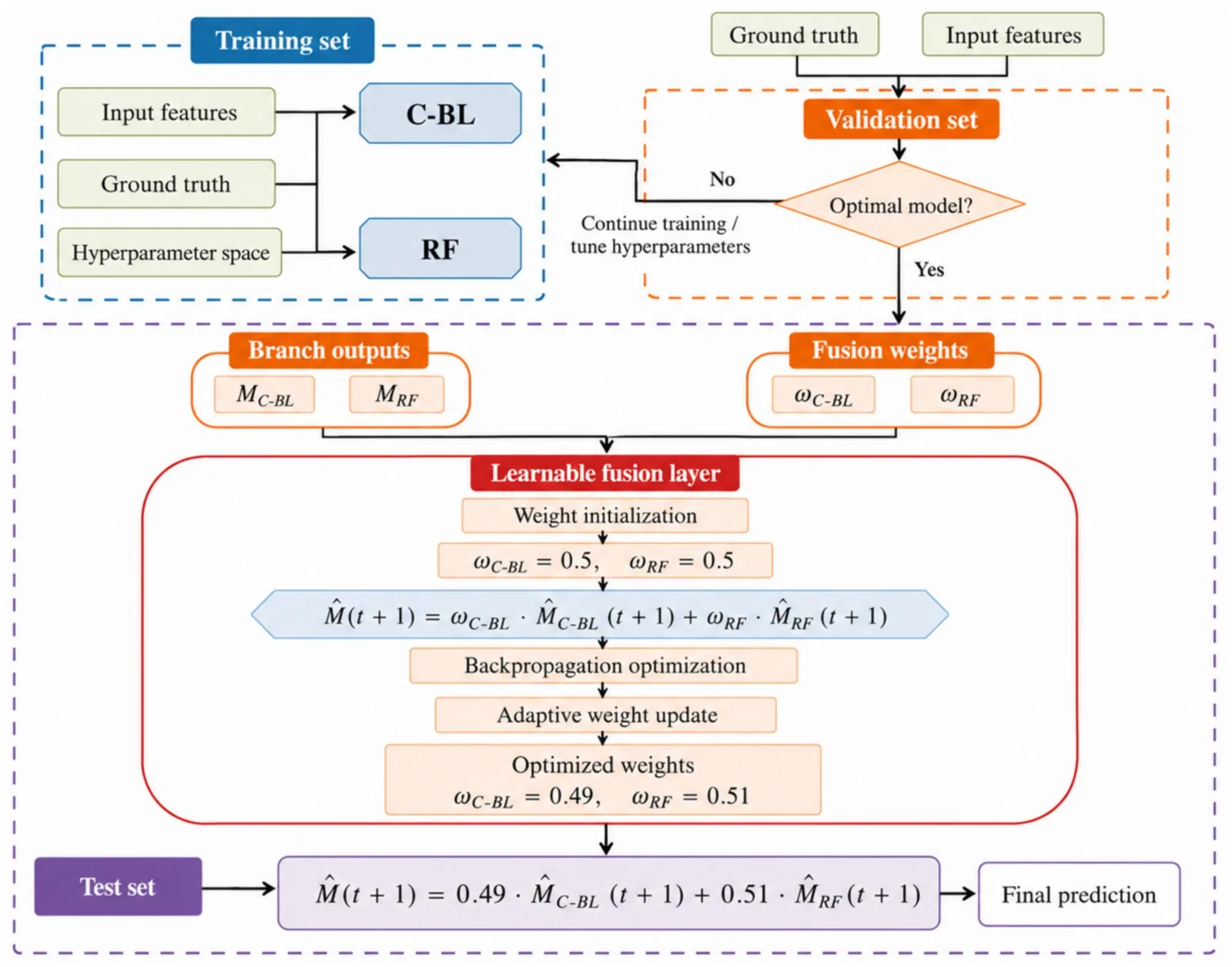
Design-decision flowchart and adaptive weighted-fusion mechanism of the proposed C-BL-R model.

Fig 7. summarizes the design rationale and workflow of the proposed C-BL-R model, including branch construction, model optimization, and adaptive fusion. During the training stage, the input features and corresponding ground-truth values are simultaneously fed into the two branches, while the hyperparameter space of each branch is optimized using MMGO to determine suitable model configurations. The validation set is subsequently employed to evaluate the model performance and guide the iterative adjustment of training parameters. After obtaining the individual predictions from the C-BL and RF branches, an adaptive learnable weighted-fusion layer is introduced to integrate the complementary information provided by the two predictors. In this framework, MMGO is employed for branch-level hyperparameter optimization, whereas the fusion weights are adaptively determined during the fusion stage based on the prediction performance of the two branches.

Unlike conventional ensemble strategies that manually determine weights according to validation errors, the proposed fusion layer treats the contribution coefficients of the two branches as trainable parameters. The initial fusion weights are set equally as ωC-BL = 0.5 and ωRF = 0.5, and they are optimized jointly with the model parameters through backpropagation by minimizing the prediction loss. The final optimized coefficients converge to ωC-BL = 0.49 and ωRF = 0.51, and this result indicates that the C-BL and RF branches provide comparable but complementary contributions to the prediction task.

#### Preliminary feature extraction layers.

Specifically, input features first pass through a Conv layer to extract local features within the sequence, followed by batch normalization to stabilize the data distribution and a ReLU activation function to introduce nonlinearity. Subsequently, a MaxPool layer performs downsampling on the features to enhance their robustness. This “Conv - BatchNorm - ReLU - MaxPool” architecture is repeated twice; ultimately, the output multi-dimensional features are flattened and concatenated to form a high-dimensional preliminary feature vector.

#### Deep feature extraction layers.

The processed features enter the BiLSTM network, which leverages its bidirectional LSTM layers to capture forward and backward dependencies within the extracted feature representations, without using information beyond prediction time t. Network weights are optimized via gradient descent to minimize the loss function. At each time step, the hidden states from both directions are concatenated to form a comprehensive feature representation, which are ultimately subjected to ReLU activation for nonlinear transformation to retain positive responses.

#### Fully connected layer.

The deeply extracted features are fed into a stacked fully connected (FC) layer, where they undergo layer-by-layer nonlinear transformations through hidden layers of unified dimensions. After multiple rounds of integration and refinement, the learned high-level abstract features ultimately yield a single-value prediction result. Table 2. details the specific parameter settings for this process.

**Table 2:** Architectural hyperparameters of the C-BL network.

Name	Layer	Filters	Kernel	Padding	output
CNN	Input (10 + 8 features)				18(S) × 1(S) × 1(C) × 1(B)
	conv_1	98	(2,1)	valid	17(S) × 1(S) × 98(C) × 1(B)
	batchnorm_1				17(S) × 1(S) × 98(C) × 1(B)
	relu_1				17(S) × 1(S) × 98(C) × 1(B)
	maxpool_1		(2,1)	valid	16(S) × 1(S) × 98(C) × 1(B)
	conv_2	96	(2,1)	valid	15(S) × 1(S) × 96(C) × 1(B)
	batchnorm_2				15(S) × 1(S) × 96(C) × 1(B)
	relu_2				15(S) × 1(S) × 96(C) × 1(B)
	maxpool_2		(2,1)	valid	14(S) × 1(S) × 96(C) × 1(B)
	flatten				1344(C) × 1(B)
BiLSTM	biLSTM	82			164(C) × 1(B)
	relu_3				164(C) × 1(B)
FC	fc	1			1(C) × 1(B)

#### Model training and hyperparameter optimization.

The detailed architecture of the C-BL component is summarized in Table 2. The CNN module consists of two convolutional layers with 98 and 96 filters, respectively. Both convolutional layers adopt a kernel size of (2,1) with same padding, followed by batch normalization, ReLU activation, and max-pooling operations to enhance local feature extraction capability from the structured input features. After the CNN-based feature extraction process, the output features are flattened and subsequently fed into the BiLSTM layer. The BiLSTM module contains 82 hidden units and is employed to capture the ordered dependencies among extracted feature representations. Finally, a fully connected layer is used to map the extracted high-dimensional features into the predicted capsizing moment output.

During model training, the stride was set to 1, and the CNN-BiLSTM network was trained for 70 epochs with a batch size of 1875. The key hyperparameters of the C-BL-R model were optimized using MMGO under identical optimization conditions. Specifically, MMGO was selected because the hyperparameter optimization problem involves both discrete structural parameters and continuous model parameters. Its adaptive exploration and exploitation mechanisms provide an effective balance between exploration and exploitation during hyperparameter optimization.

In this study, MMGO was employed to optimize the hyperparameters of the C-BL and RF branches. The predefined parameter spaces included network configurations and hidden-layer settings for the C-BL branch, and the number of decision trees and minimum leaf size for the RF branch. Candidate configurations were evaluated using the validation-set MAE, and the configuration with the lowest MAE was selected. The fusion coefficients were then optimized through the adaptive fusion mechanism.

The final RF component adopted 200 decision trees and a minimum leaf size of 1. To improve the reliability of the optimization process, five additional runs with different random initializations were conducted after the best-performing parameter combination was identified, and the final model configuration was determined based on the repeated optimization results.

## Dataset and preprocessing

### Dataset

The CFD-generated dataset used in this study consists of 10 input features and one target output. For each shiplift model, all hydrodynamic responses were obtained from a single ANSYS Fluent simulation. The transverse and longitudinal capsizing moments at the bottom of the chamber, together with the corresponding force responses, were extracted separately from the same simulation results. The seismic acceleration input was applied simultaneously in the transverse, longitudinal, and vertical directions as a unified excitation condition. The transverse and longitudinal response histories were not combined into a two-dimensional feature matrix. Instead, they were converted into independent samples and jointly used to train the unified C-BL-R model. Specifically, the geometric parameters [52] are direction-independent, while the dynamic response variables correspond to the specific prediction direction of each sample. Therefore, each sample contains the historical response information associated with one direction and the corresponding target capsizing moment at the next sampling instant.

For each shiplift model, 500 transverse samples and 500 longitudinal samples were generated, resulting in 1000 samples per case. Finally, the 19 independent shiplift models produced 19,000 samples for model training and evaluation. All 19,000 samples in the model-development dataset were generated using the El-Centro record scaled to a PGA of 0.1g. The Taft and Koyna records were completely excluded from the training, validation, and internal test subsets and were used only for external validation. No synthetic samples or artificially generated capsizing-moment targets were introduced; all input variables and target responses were directly extracted from CFD-generated response histories. The samples from all shiplift cases were randomly divided into training, validation, and test subsets. Because case-specific experimental measurements of capsizing moments are unavailable for the 19 investigated configurations, the proposed surrogate model is developed to approximate the CFD-based response relationship rather than directly reproduce experimentally measured full-scale responses.

### Preprocessing

The transient CFD simulations may introduce small high-frequency numerical fluctuations in the capsizing moment histories due to discretization errors and iterative convergence processes. Therefore, a smoothing procedure was applied in MATLAB to reduce insignificant numerical fluctuations while preserving the overall trend and peak characteristics of the capsizing moment. Since the input variables have different physical dimensions and numerical ranges, Z-score normalization was subsequently performed before model training to improve numerical stability and ensure comparable contributions of different features during optimization. The formulation is given as follows:


$$\begin{array}{c} z_{i}=\frac{x_{i}-\mu }{\sigma } \end{array}$$ (19)

where $x_{i}$ denotes the *i*-th sample in the dataset, μ and σ represent the mean and standard deviation of the corresponding feature, respectively, and $z_{i}$ is the normalized value.

Following normalization, eight additional features were generated from the 10 original physical variables using CNN-based feature enhancement. These features were concatenated with the original variables to form an 18-dimensional input representation, which was subsequently fed into both the C-BL and RF branches. The feature-enhancement CNN serves as a preprocessing module and is different from the CNN component in the C-BL branch. Subsequently, all samples from the 19 shiplift cases were randomly shuffled and partitioned into training, validation, and test sets with a ratio of 8:1:1.

### Evaluation metrics

To compare the prediction performance of different models on the capsizing moment at the bottom of the ship chamber, five evaluation metrics are adopted in this study [53,54]: Mean Absolute Error (MAE), Mean Absolute Percentage Error (MAPE), Mean Squared Error (MSE), Root Mean Squared Error (RMSE), and Coefficient of Determination (R^2^). Specifically, an R^2^ value approaching 1 indicates a higher predictive accuracy. The calculation formulas for each metric are as follows:


$$\begin{array}{c} MAE=\frac{\text{1}}{n}\sum_{i=\text{1}}^{n}|y_{i}-{\hat{y}}_{i}| \end{array}$$ (20)

$$\begin{array}{c} MAPE=\frac{\text{100}\%}{n}\sum_{i=\text{1}}^{n}\left|\frac{y_{i}-{\hat{y}}_{i}}{y_{i}}\right| \end{array}$$ (21)

$$\begin{array}{c} MSE=\frac{\text{1}}{n}\sum_{i=\text{1}}^{n}{\left(y_{i}-{\hat{y}}_{i}\right)}^{\text{2}} \end{array}$$ (22)

$$\begin{array}{c} RMSE=\sqrt{\frac{\text{1}}{n}\sum_{i=\text{1}}^{n}{\left(y_{i}-{\hat{y}}_{i}\right)}^{\text{2}}} \end{array}$$ (23)

$$\begin{array}{c} R^{\text{2}}=\text{1}-\frac{\sum_{i=\text{1}}^{n}(y_{i}-{\hat{y}}_{i}{)}^{\text{2}}}{\sum_{i=\text{1}}^{n}(y_{i}-\bar{y}{)}^{\text{2}}} \end{array}$$ (24)

where $y_{i}$ and ${\hat{y}}_{i}$ are the actual and predicted values for the *i*-th sample, respectively; $\bar{y}$ denotes the mean of all actual values; and *n* is the total number of samples.

## Results and discussions

### Verification of 3D CFD model for chamber-water-ship system

**Fig 8:**
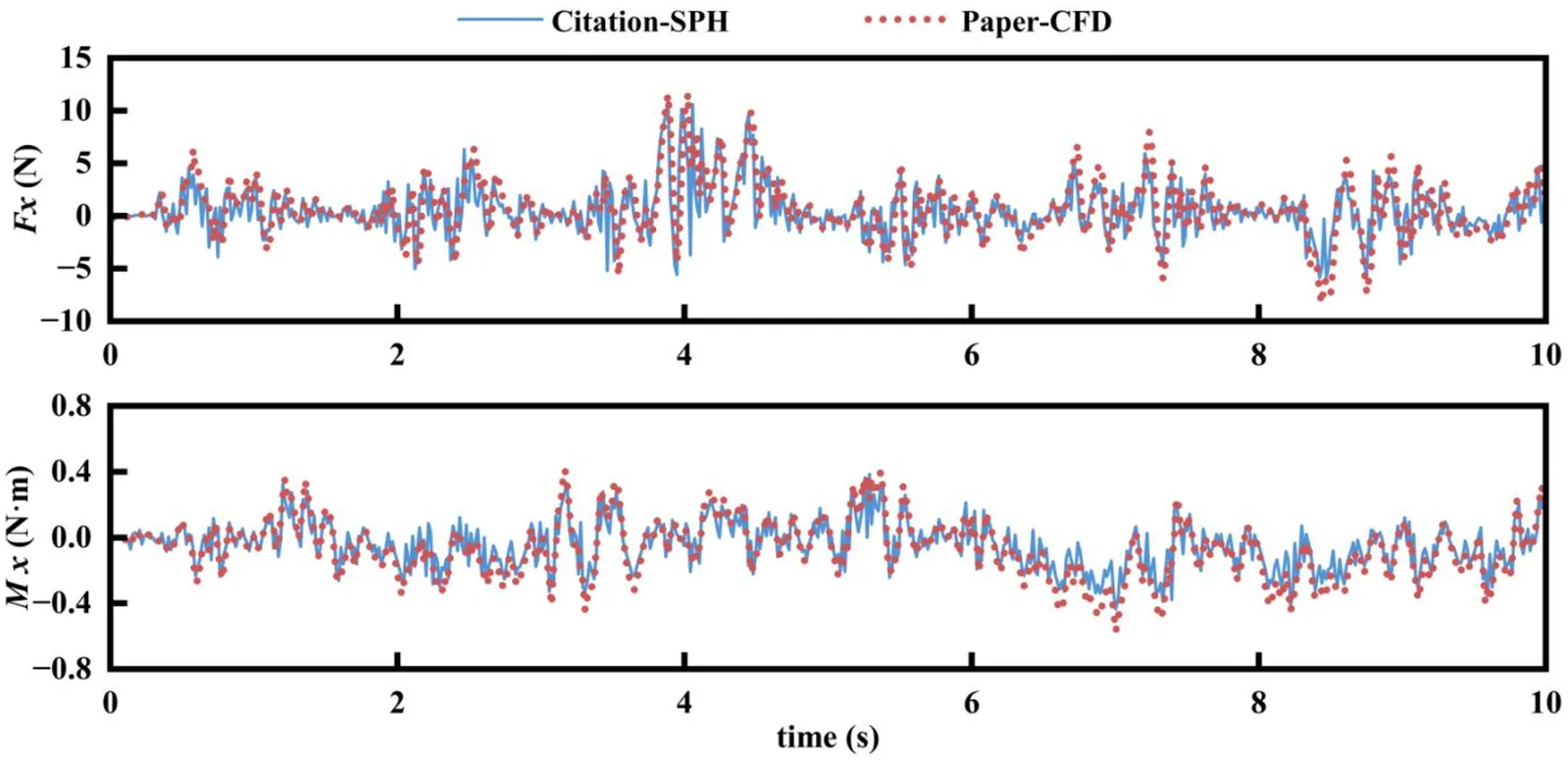
Comparison of longitudinal capsizing force and longitudinal capsizing moment predicted by the SPH method and the present CFD method under identical model conditions.

To evaluate the capability of the CFD framework adopted in this study for capturing the hydrodynamic response characteristics of the chamber-water-ship system, the 3D model from Ref. [55] was selected as a benchmark case, and the CFD results were compared with the corresponding SPH results, as shown in Fig 8.

The results show that the longitudinal capsizing force *Fx* and capsizing moment *Mx* obtained using the CFD method in this study generally follow the same trend as the results obtained using the SPH method in the reference [55]. Specifically, at t = 4s, the peak capsizing forces from the two methods are 10.92N and 10.59N, with a relative difference of about 3%. At t = 3s, the maximum capsizing moments are 0.51N·m and 0.49N·m, with a relative difference of about 4.1%. The CFD and SPH results exhibit consistent time-history trends for the examined benchmark configuration. The relative differences between the two methods in the selected peak force and peak moment are approximately 3.0% and 4.1%, respectively. This agreement provides additional confidence in the numerical framework under the examined benchmark conditions. However, this benchmark comparison is intended to provide numerical cross-validation of the CFD framework rather than experimental validation of the 19 full-scale configurations, because both datasets are numerical and the benchmark case is independent of the surrogate-model database. The CFD responses are therefore used as numerical reference data for surrogate-model development and evaluation.

**Fig 9:**
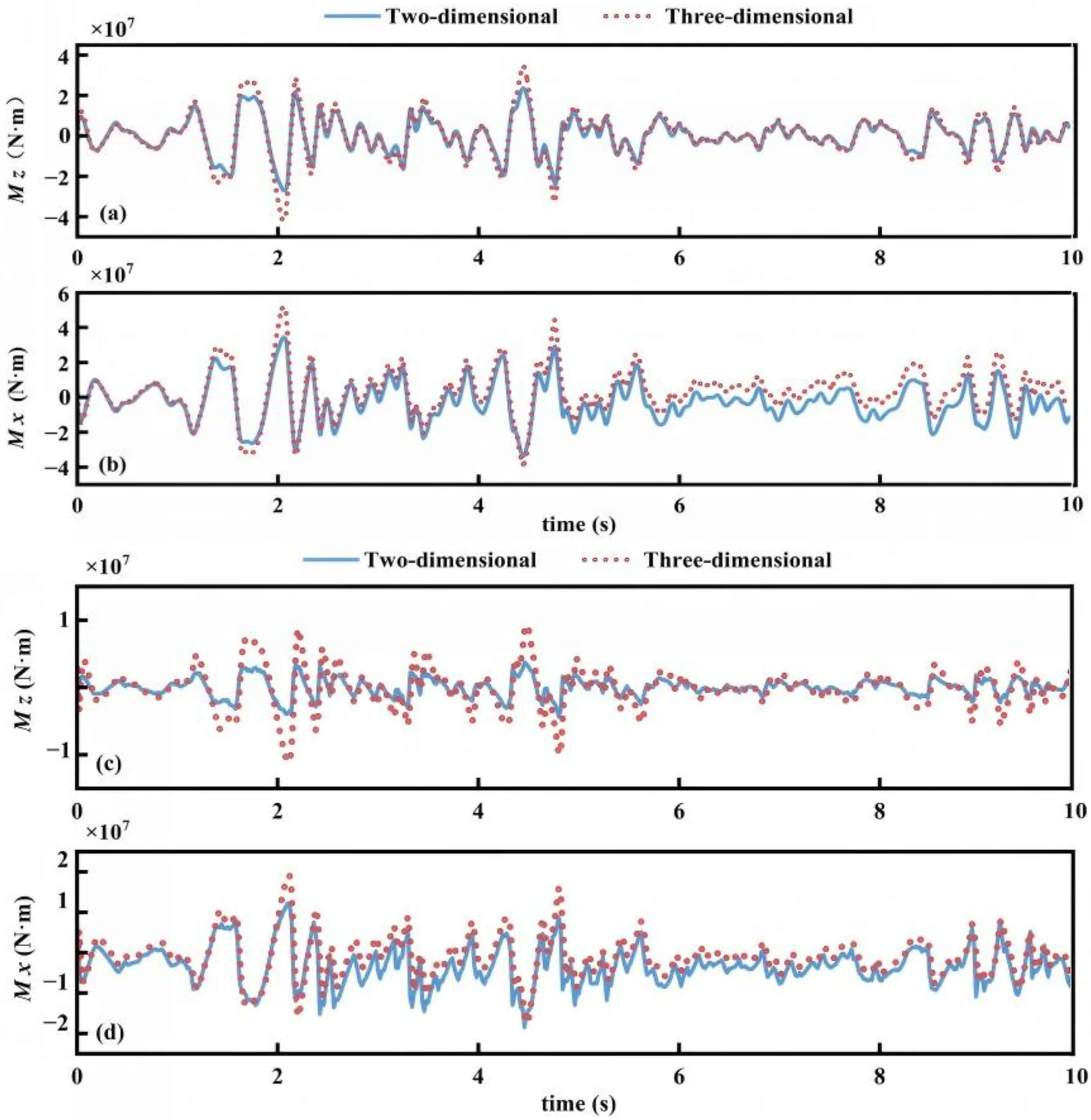
Comparison of capsizing moments predicted by the 2D and 3D models under identical seismic excitation: (a) transverse and (b) longitudinal responses for the 3000t light-load case; (c) transverse and (d) longitudinal responses for the 1350t full-load case.

To further evaluate the influence of dimensional simplification on capsizing moment prediction, the numerical results obtained from the 2D simplified model were compared with those from the 3D CFD model. Considering that shiplifts with different scales and loading conditions may exhibit different discrepancies between 2D and 3D simulations, two independent engineering cases were investigated, including the 3000t light-load condition and the 1350t full-load condition. The corresponding 2D results were calculated using the modified Housner model proposed in Ref. [56] to ensure consistency with the benchmark comparison. The comparison results are presented in Fig 9.

As shown in Fig 9, both the 2D and 3D models capture the overall variation trend of transverse and longitudinal capsizing moments under identical seismic excitation. However, noticeable differences are observed in the transient peak responses. Taking the 3000t light-load condition (Fig 9(a)-Fig 9(b)) as an example, the longitudinal capsizing moment predicted by the 3D model is higher than that obtained from the 2D model, with the maximum deviation exceeding 27% at the peak response stage. This difference is mainly attributed to the simplified representation of three-dimensional free-surface deformation and spatial fluid motion in the 2D model, which cannot fully capture the complex chamber-water-ship coupling effects. Similar behavior is also observed in the additional 1350t full-load condition (Fig 9(c)-Fig 9(d)). The peak longitudinal capsizing moment predicted by the 3D model is approximately 30% higher than that from the 2D model. The consistent trend observed in the two examined cases suggests that the 2D simplification may underestimate the peak longitudinal capsizing moment under different scales and loading conditions. However, the magnitude of the discrepancy remains case-dependent. Since the longitudinal capsizing moment is a critical indicator for seismic safety assessment of shiplift structures, these comparisons highlight the importance of considering three-dimensional hydrodynamic effects when evaluating extreme dynamic responses.

### Comparative evaluation of the proposed C-BL-R model with benchmark models

To evaluate the prediction stability and generalization performance of the proposed C-BL-R model, five independent training runs were conducted, with the full dataset randomly repartitioned into training, validation, and test sets at an 8:1:1 ratio for each run. The prediction performance on the Training set, Validation set, and Testing set was evaluated using five commonly adopted metrics, including MAE, MAPE, MSE, RMSE, and R^2^. Considering the stochastic nature of model training, all results are presented in the form of mean ± standard deviation (Mean±SD), as summarized in Table 3.

**Table 3:** Prediction performance of the proposed C-BL-R model on the training, validation, and test set.

Dataset	MAE	MAPE	MSE	RMSE	R2
Training	0.0471 ± 0.0007	0.705 ± 0.0600	0.0067 ± 0.0002	0.0817 ± 0.0014	0.9822 ± 0.0008
Validation	0.0775 ± 0.0019	0.873 ± 0.0750	0.0218 ± 0.0022	0.1475 ± 0.0072	0.9405 ± 0.0042
Test	0.0761 ± 0.0024	1.110 ± 0.3360	0.0201 ± 0.0038	0.1408 ± 0.0128	0.9466 ± 0.0065

As shown in Table 3, the proposed C-BL-R model maintained stable predictive performance in the training set, validation set, and test set. The model achieved the lowest prediction error on the training set, with MAE of 0.0471 ± 0.0007, RMSE of 0.0817 ± 0.0014, and R^2^ value of 0.9822 ± 0.0008, indicating that the model effectively captures the nonlinear relationship between input variables and capsizing moment response. For the test set, the MAE of the model is 0.0761 ± 0.0024, the RMSE is 0.1408 ± 0.0128, and the R^2^ value is still as high as 0.9466 ± 0.0065. Although the prediction error slightly increases compared to the training set, the performance degradation is limited. Meanwhile, the increase in MAPE is considered reasonable because MAPE is highly sensitive to small capsizing moment responses, and small absolute deviations may lead to relatively large percentage errors. These results indicate that no severe overfitting tendency was observed, and the proposed C-BL-R model has reliable generalization ability.

To further validate the superiority of the proposed C-BL-R model, we selected seven benchmark models including C-BL, C-LSTM, BiLSTM-RF (BL-R), CNN, BiLSTM, LSTM, and RF for comparative analysis. All models were evaluated under the same experimental conditions, and their predictive performance on the test set is summarized in Table 4.

**Table 4:** Comparison of predictive performance of different machine learning models on the test set.

Model	MAE	MAPE	MSE	RMSE	R2
C-BL-R	0.0761 ± 0.0024	1.110 ± 0.3360	0.0201 ± 0.0038	0.1408 ± 0.0128	0.9466 ± 0.0065
C-BL	0.0803 ± 0.0019	0.944 ± 0.1810	0.0240 ± 0.0028	0.1548 ± 0.0297	0.9381 ± 0.0052
C-LSTM	0.0878 ± 0.0038	0.872 ± 0.2610	0.0296 ± 0.0035	0.1720 ± 0.0191	0.9295 ± 0.0049
BL-R	0.0833 ± 0.0023	1.119 ± 0.4770	0.0239 ± 0.0022	0.1545 ± 0.0370	0.9404 ± 0.0055
CNN	0.0930 ± 0.0041	1.653 ± 0.3950	0.0246 ± 0.0025	0.1568 ± 0.0182	0.9411 ± 0.0041
BiLSTM	0.0848 ± 0.0035	1.097 ± 0.2540	0.0219 ± 0.0019	0.1481 ± 0.0266	0.9402 ± 0.0063
LSTM	0.0850 ± 0.0026	0.872 ± 0.1480	0.0224 ± 0.0023	0.1498 ± 0.0174	0.9379 ± 0.0032
RF	0.0827 ± 0.0039	1.341 ± 0.2830	0.0253 ± 0.0031	0.1591 ± 0.0285	0.9270 ± 0.0035

As shown in Table 4, the proposed C-BL-R model achieved the best comprehensive performance in terms of MAE, MSE, RMSE, and R^2^ on the test set, with the lowest MAE (0.0761 ± 0.0024), MSE (0.0201 ± 0.0038), and RMSE (0.1408 ± 0.0128), as well as the highest R^2^ value (0.9466 ± 0.0065). These results demonstrate its superior generalization capability for capsizing moment prediction. Among the benchmark models, C-BL and C-LSTM exhibited relatively competitive performance, while their prediction errors remained higher than those of C-BL-R, indicating the effectiveness of introducing the RF component for nonlinear regression enhancement. BL-R and RF achieved reasonable accuracy but showed lower R^2^ values on the test set, suggesting weaker generalization compared with the proposed model. The individual neural network models (CNN, BiLSTM, and LSTM) showed relatively inferior performance, as they lack the capability to simultaneously capture local features and complex nonlinear relationships. Overall, the integration of CNN, BiLSTM, and RF enables C-BL-R to achieve more comprehensive feature extraction and more accurate prediction.

### Effects of different parameters on the predictive accuracy of C-BL-R model

Three model-design factors were examined in this section: neuron number, outlier-detection method, and optimization algorithm. A sequential one-factor-at-a-time sensitivity analysis was adopted, in which one factor was varied while the remaining settings were fixed at the values used in that stage. This procedure was intended to compare the conditional influence of the investigated candidates under a manageable computational budget, rather than to perform an exhaustive joint optimization of all interacting components. Therefore, the selected configuration should be interpreted as the best-performing empirical configuration among the evaluated candidates and along the adopted search path, not as a mathematically guaranteed global optimum. The evaluation metrics included MAE, RMSE, and R^2^. Based on the comparison results, the configuration with the best predictive performance among the evaluated candidates was selected for the final C-BL-R model.

#### Effects of different neuron counts.

**Fig 10:**
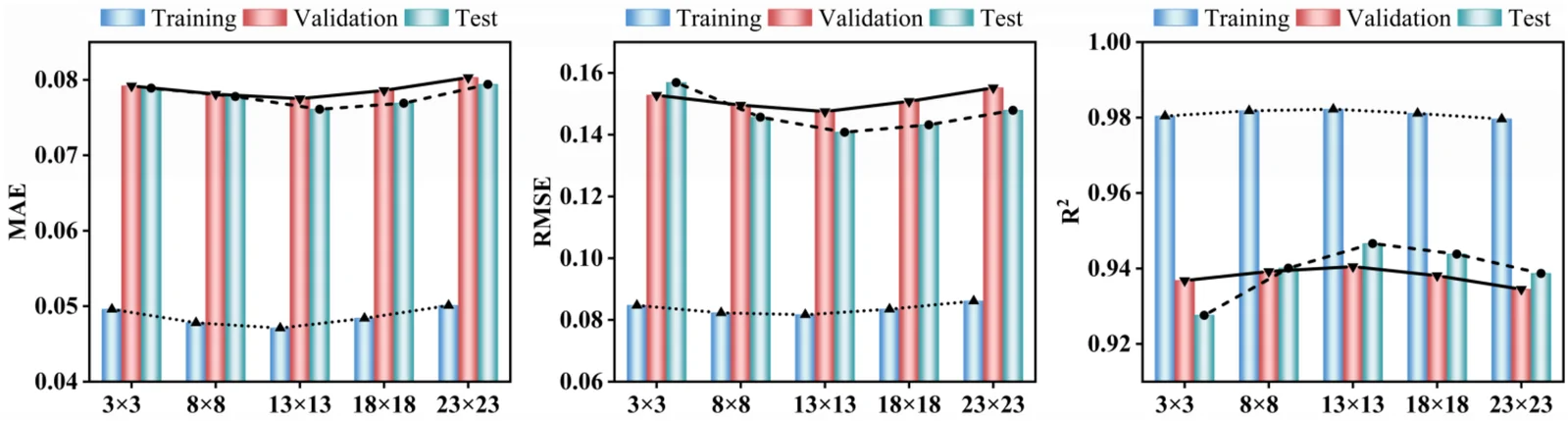
Comparison of prediction performance metrics among five different neuron numbers including 3 × 3.

Existing studies [57] have demonstrated that an inappropriate number of neurons may negatively affect neural network performance. An excessive number of neurons increases model complexity and may result in overfitting, whereas an insufficient number limits the representation capability and causes underfitting. Therefore, the optimal neuron number of the C-BL-R model was first determined within the range of 3–23 according to empirical formulations such as the Kolmogorov formula [54,58]. Five representative neuron configurations (3 × 3, 8 × 8, 13 × 13, 18 × 18, and 23 × 23) were selected at equal intervals to investigate the influence of neuron number on model prediction performance. The corresponding results are presented in Fig 10.

As illustrated in Fig 10, the five neuron configurations exhibit comparable performance on the training set, indicating that all structures possess sufficient fitting capability. For the validation set, the 13 × 13 neuron configuration obtains the best performance, with MAE, RMSE, and R2 values of 0.07750, 0.14750, and 0.94050, respectively. Compared with the 8 × 8 configuration, the 13 × 13 structure slightly reduces prediction errors and improves the coefficient of determination.

On the test set, the 13 × 13 configuration also achieves the highest prediction accuracy, with MAE, RMSE, and R2values of 0.07610, 0.14080, and 0.94660, respectively. However, further increasing the neuron number to 18 × 18 and 23 × 23 results in increased errors and decreased R2, indicating that excessive neurons may introduce redundant parameters and weaken model generalization. Therefore, the 13 × 13 neuron configuration is selected as the optimal structure.

#### Effects of different outlier detection methods.

**Fig 11:**
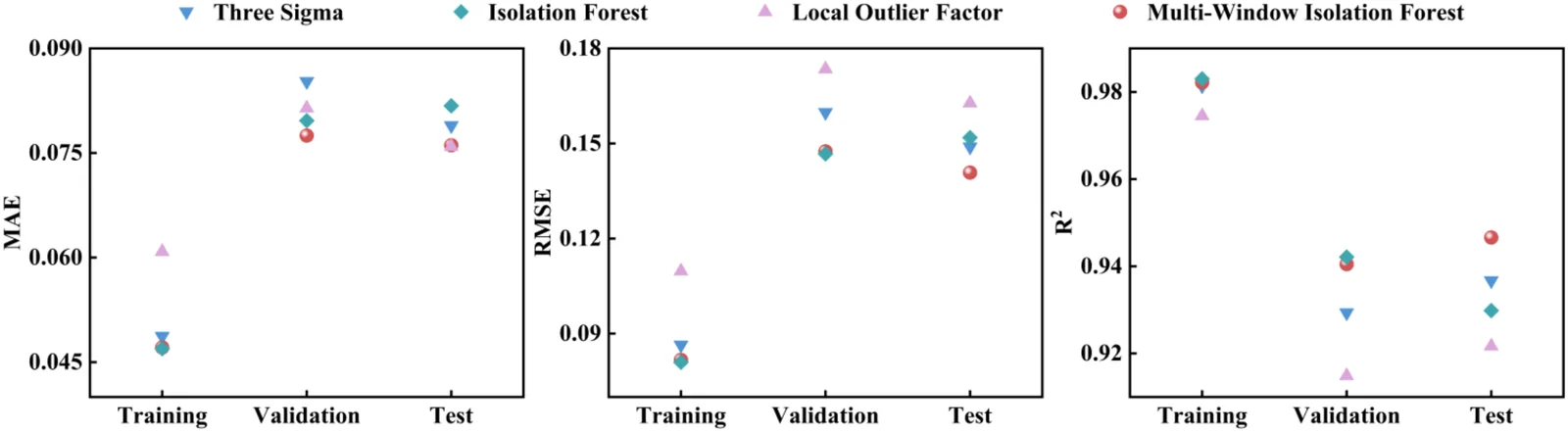
Comparison of prediction performance among four different outlier detection methods: Three Sigma, Isolation Forest, Local Outlier Factor, and Multi-Window Isolation Forest.

Outlier detection, which optimizes data quality by identifying and handling abnormal samples, is a crucial preprocessing step for enhancing the generalization ability of the C-BL-R model. To systematically evaluate the impact of different outlier detection methods on the performance of the C-BL-R model, this study selected four representative algorithms for comparison: Three Sigma, Isolation Forest, Local Outlier Factor (LOF), and Multi-Window Isolation Forest (MWIF). The results are illustrated in Fig 11.

As illustrated in Fig 11, the prediction performance obtained with different outlier detection methods shows noticeable differences among the training set, validation set, and test set. On the training set, Three Sigma, MWIF, and Isolation Forest exhibit relatively similar performance, whereas LOF produces larger prediction errors, with MAE and RMSE values of 0.06084 and 0.10972, respectively.

On the validation set, MWIF and Isolation Forest achieve lower error values than the other two methods. However, considering the overall performance on unseen samples, MWIF provides a more balanced prediction capability. Specifically, on the test set, MWIF achieves an MAE of 0.07610, an RMSE of 0.14080, and an R2 of 0.94660, outperforming Three Sigma and Isolation Forest in terms of comprehensive prediction accuracy. Although LOF obtains a slightly lower MAE value (0.07592), its RMSE increases to 0.16273 and R2 decreases to 0.92165, indicating unstable prediction performance. In addition, the R2 of Isolation Forest decreases from 0.98301 on the training set to 0.92984 on the test set, suggesting reduced generalization ability. With the neuron configuration and remaining settings fixed, MWIF was selected from the four evaluated outlier-detection methods according to the predefined validation criterion and was used in the subsequent comparison.

#### Effects of different optimization algorithms.

**Fig 12:**
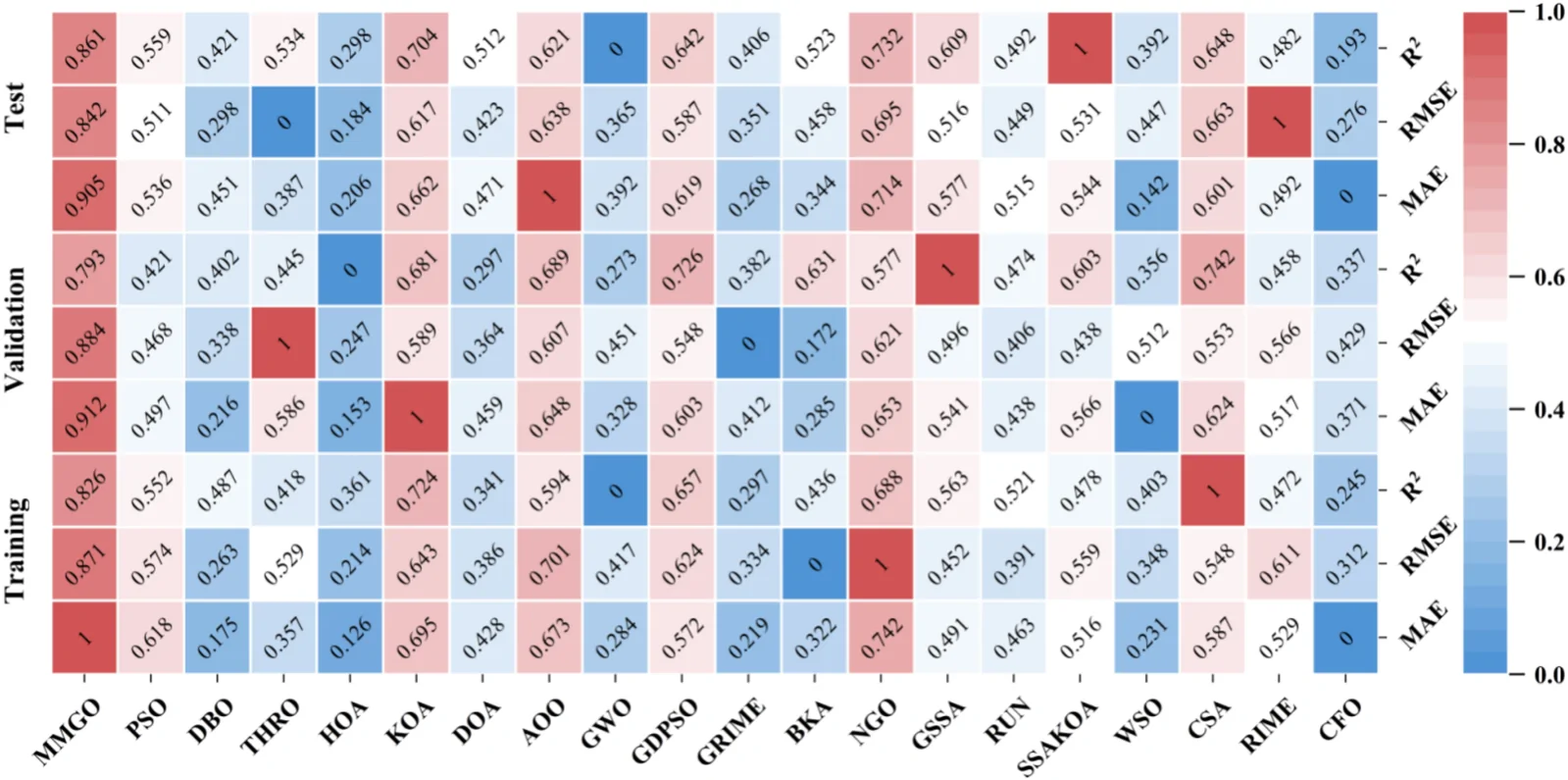
Comparison of prediction performance among twenty different optimization algorithms, including MMGO and PSO.

Optimization algorithms play an important role in improving the searching efficiency and convergence performance of the C-BL-R model by optimizing model parameters and minimizing the defined objective function. To investigate the influence of different optimization strategies on model performance, twenty representative metaheuristic optimization algorithms were selected (S1 Table). These algorithms include classical algorithms: particle swarm optimization (PSO), grey wolf optimizer (GWO), established metaheuristic algorithms: dung beetle optimizer (DBO), northern goshawk optimization (NGO), runge kutta optimizer (RUN), white shark optimizer (WSO), kepler optimization algorithm (KOA), rime ice-based optimization algorithm (RIME), hiking optimization algorithm (HOA), Chameleon Swarm Algorithm (CSA), golden sine particle swarm optimization (GDPSO), golden sine and mapping improved sparrow search algorithm (GSSA), and emerging algorithms proposed in recent years: mapping·mountain·gazelle·optimizer (MMGO) [59], black-winged kite algorithm (BKA), animated oat optimization algorithm (AOO), caterpillar fungus optimizer (CFO), tianji’s horse racing optimization (THRO), salp swarm-kepler optimization algorithm (SSAKOA), dhole optimization algorithm (DOA), generalized rime ice-based optimization algorithm (GRIME). All metrics were normalized to the [0, 1] interval, with a value of 1 indicating optimal performance. Fig 12. presents the results.

As shown in Fig 12, under the fixed network and preprocessing settings and the common computational budget adopted in this comparison, MMGO achieved the best validation performance among the investigated algorithms for this specific C-BL-R hyperparameter-optimization task. On the test set, MMGO obtains MAE, RMSE, and R2 values of 0.905, 0.842, and 0.861, respectively, demonstrating its competitive prediction accuracy and stability. Although several algorithms, such as KOA, NGO, and SSAKOA, exhibit competitive performance in some individual metrics, their overall performance remains inferior to MMGO. In contrast, algorithms such as GWO and CFO show relatively weaker optimization capability, resulting in lower prediction performance of the C-BL-R model. Therefore, MMGO was therefore selected for the final model within the scope of the evaluated candidates.

### Case analysis

To provide an additional external evaluation beyond the randomly partitioned dataset, four operating conditions based on one chamber geometry that was not included in the 19-case model-development dataset were investigated. These cases were established based on the preliminary design scheme of the 3000 t ship chamber developed within the National Key Research and Development Program “Complete Technology of 200 m-scale Large Vertical Shiplift”. All cases adopted the same ship chamber geometry with dimensions of 115m × 18.4m × 10.5m (length×width×height). The investigated conditions included a 3000 t full-load ship, a 3000 t light-load ship, a 1000 t full-load ship, and a ship-absence case. The 3000 t full-load and light-load ships shared the same hull geometry (88.0m × 16.3m × 6.48m) but differed in loading state, while the 1000 t full-load ship had dimensions of 67.5m × 10.8m × 3.7m.

**Fig 13:**
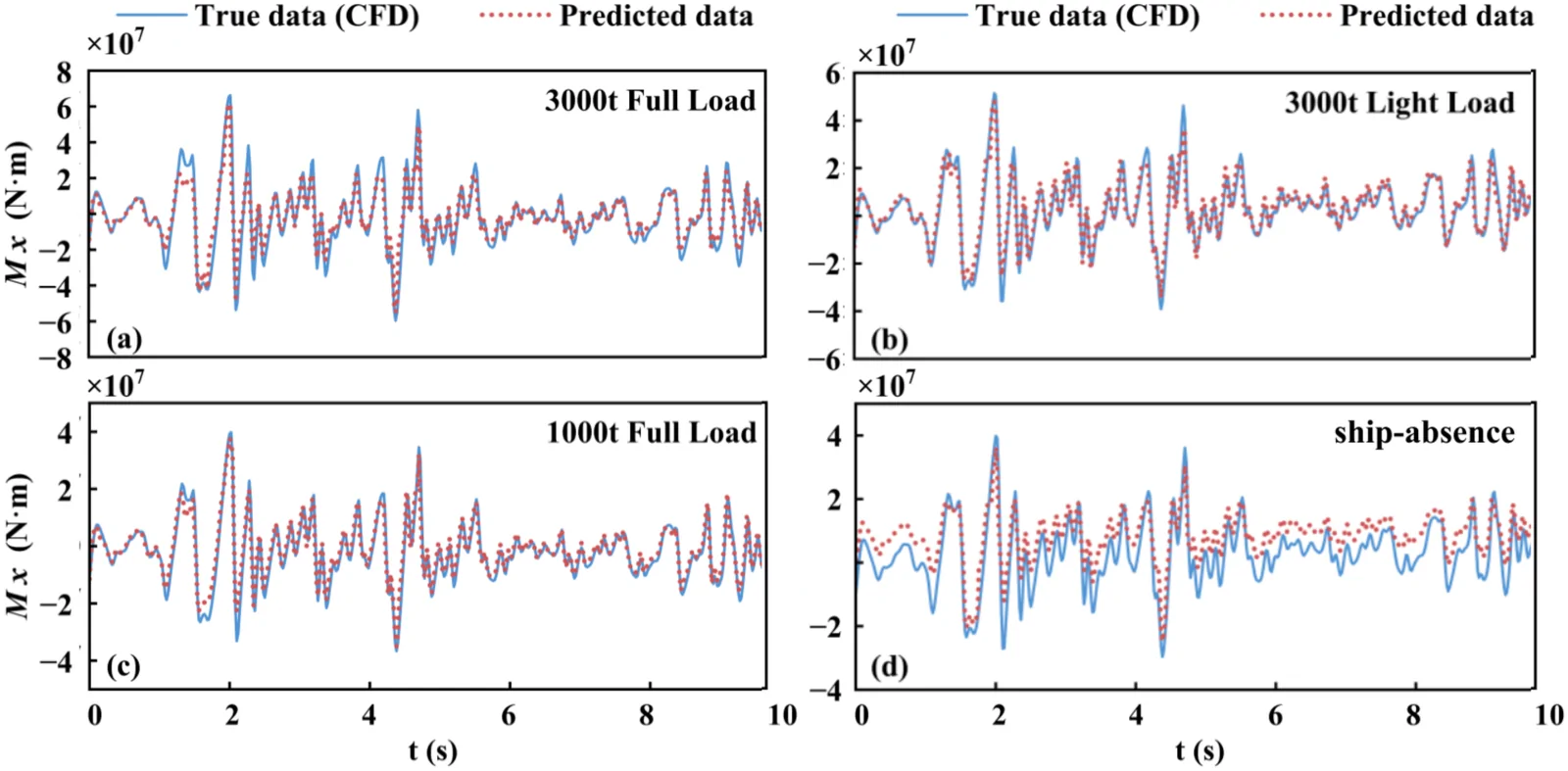
Comparison of longitudinal capsizing moment results between CFD and C-BL-R models under four conditions: (a) 3000t Full Load, (b) 3000t Light Load, (c) 1000t Full Load, and (d) ship-absence.

The CFD simulations of these four cases were completely independent from the training set and were used solely for evaluating the predictive capability of the proposed surrogate model under unseen operating conditions. Under identical external boundary conditions, the C-BL-R model performed sequential one-step-ahead prediction of the longitudinal capsizing moment based on the available input variables at each prediction instant t. Specifically, four geometric parameters (L2/L1, B2/B1, H2/H, H/H1) and six historical dynamic response variables, including *a*(t − 1), *a*(t), *F*(t − 1), *F*(t), *M*(t − 1), and *M*(t), were used as inputs to predict the capsizing moment at the next time step *M*(t + 1). Therefore, all input variables were available at or before the current prediction instant, and no future response information was involved. For the ship-absence validation cases, the ship-related geometric inputs L2/L1, B2/B1, and H2/H were set to zero, while H/H1 was determined from the actual chamber geometry and water level. The CFD-generated response histories provide the historical states required for one-step-ahead prediction and the reference target values for evaluating model accuracy. The predicted value *M*(t + 1) was not recursively used as *M*(t) for the next prediction step; instead, each prediction used the corresponding observed CFD-derived historical states. The comparison between CFD simulations and C-BL-R predictions is presented in Fig 13.

As shown in Fig 13., the predicted capsizing moment histories from the C-BL-R model generally agree well with the CFD results under the four independent operating conditions. The model generally captures the overall variation trends and major peak responses without significant phase shifts. For the ship-present cases, the 3000t full-load condition exhibited the largest peak capsizing moment, reaching 6.6 × 107N·m, while the corresponding prediction by the C-BL-R model was 6.3 × 107N·m, with a deviation of 4.8%. The 1000t full-load condition showed the smallest peak value of 3.96 × 107N·m, and the predicted value was 3.79 × 107N·m, corresponding to a deviation of 4.5%. These results demonstrate that the proposed model maintains reliable one-step-ahead prediction capability for unseen ship-present operating conditions.

However, under the ship-absence case, although the C-BL-R model can capture the overall evolution trend of the capsizing moment, the prediction deviation increases to approximately 12.8%, which is higher than that observed in ship-present cases (below 4.8%). This increased deviation is mainly attributed to the absence of ship-absence samples in the training dataset. Since all 19 shiplift cases and approximately 19,000 samples used for training were generated under ship-present conditions, the model had limited capability to generalize to this unseen condition. In addition, the absence of the ship changes the hydrodynamic response characteristics within the chamber, further increasing the prediction deviation. Therefore, the current model should be applied primarily to ship-present operating conditions and is not recommended for quantitative prediction under ship-absence conditions without further retraining and validation. If the model is intended to be applied to ship-absence scenarios, additional ship-absence cases should be incorporated, followed by retraining and independent validation.

**Fig 14:**
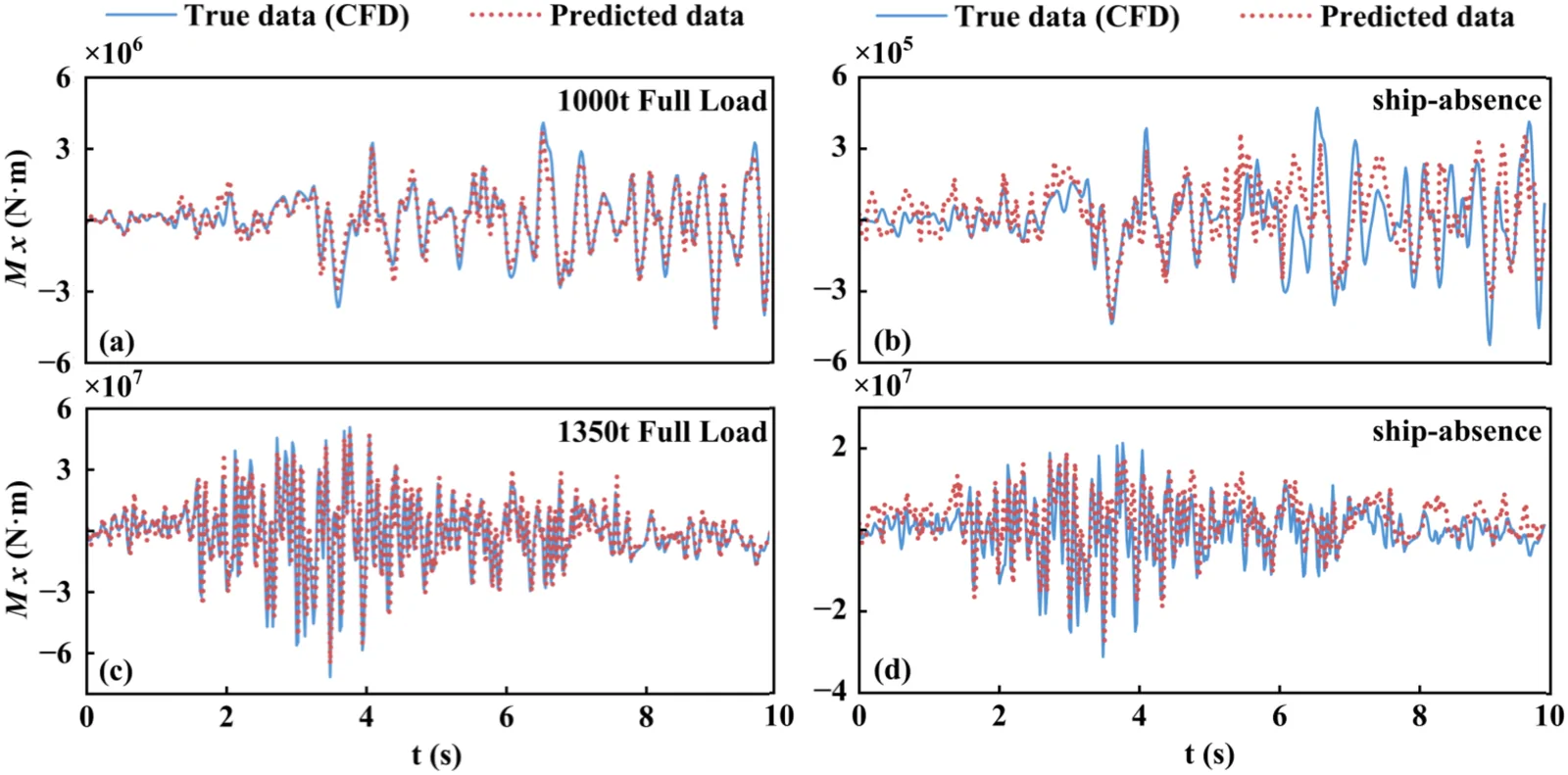
Comparison of longitudinal capsizing moment results between CFD and C-BL-R models under unseen seismic excitations: (a) 6-degree Taft earthquake with 1000t Full Load and (b) ship-absence; (c) 8-degree Koyna earthquake with 1350t Full Load and (d) ship-absence.

To evaluate the external predictive performance of the proposed C-BL-R model under seismic records not included in model development, two groups of additional CFD validation cases were introduced. The Taft record scaled to a PGA of 0.05g was applied to the 1000 t full-load Niederfinow shiplift, whereas the Koyna record scaled to a PGA of 0.2g was applied to the 1350 t full-load Lokeren shiplift. For each record, both ship-present and ship-absence conditions were considered. These records and cases were excluded from training, validation, internal testing, and hyperparameter optimization. The CFD simulations were performed independently, and the obtained capsizing moment responses were used as reference solutions for comparison with the C-BL-R predictions, as shown in Fig 14.

As shown in Fig 14, the C-BL-R model successfully captures the overall variation characteristics of the longitudinal capsizing moment under different seismic excitation types and intensity levels. For the full-load conditions, the predicted responses under both Taft and Koyna earthquake excitations exhibit good agreement with the CFD results, with consistent oscillation characteristics and peak response trends. These results demonstrate that the proposed model can effectively extract the nonlinear relationship between seismic excitation characteristics and shiplift dynamic responses, indicating good generalization capability beyond the seismic conditions included in the training set.

Nevertheless, under the ship-absence cases, although the predicted responses maintain consistent overall trends with the CFD results, relatively larger amplitude discrepancies are observed. The maximum deviation of the predicted peak capsizing moment reaches approximately 14%, which is higher than that under the full-load conditions. This result is consistent with the previous case analysis, indicating that additional ship-absence samples should be incorporated into the training dataset to improve the model’s generalization capability for such operating conditions.

### Model interpretability analysis

**Fig 15:**
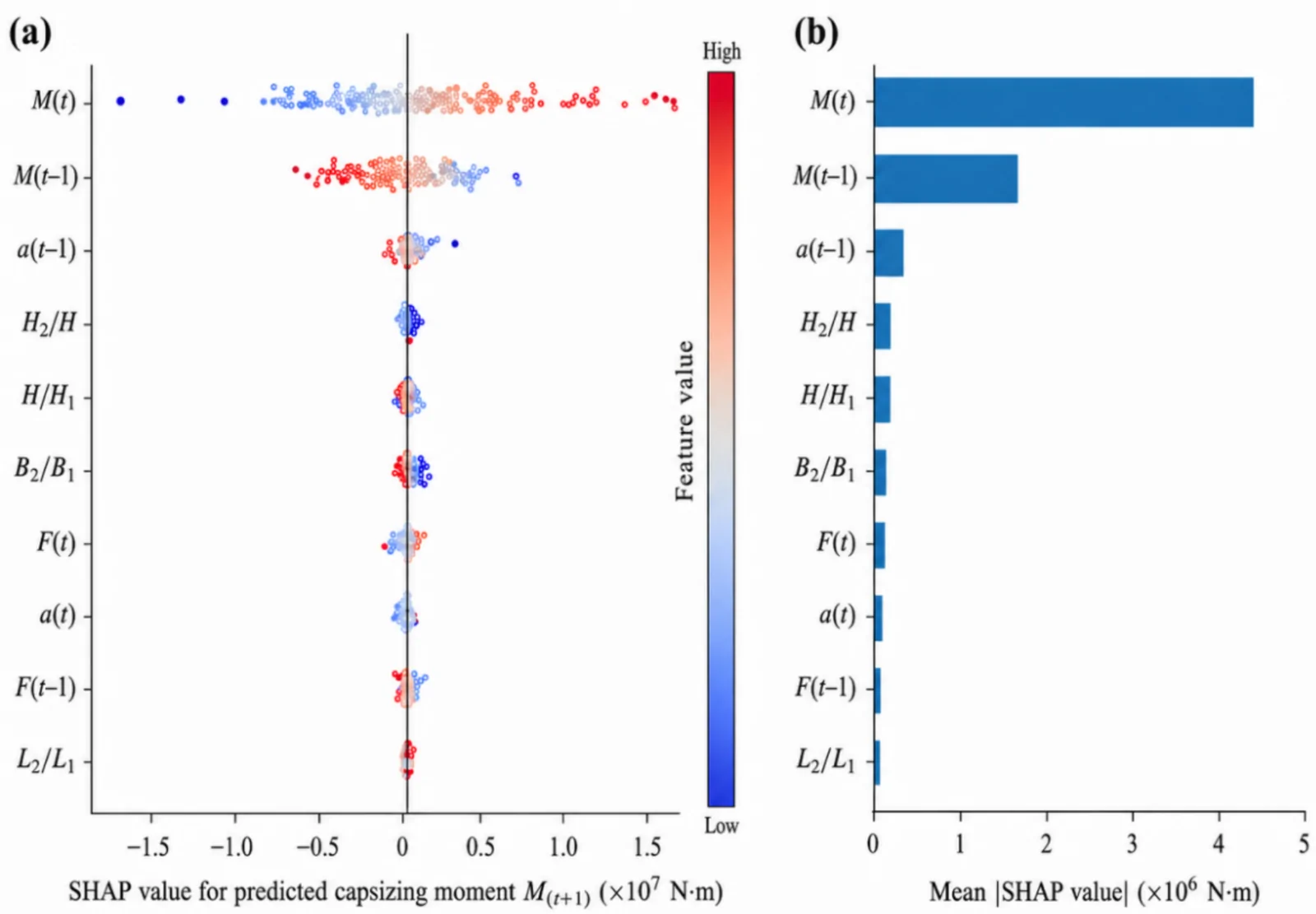
SHAP-based interpretability analysis of the C-BL-R model: (a) SHAP value distribution of the 10 input features; (b) feature importance ranking based on the mean absolute SHAP values.

To improve the interpretability of the proposed model, the SHAP (SHapley Additive exPlanations) method was employed to perform global feature importance analysis and quantify the contribution of each of the 10 input features to the predicted capsizing moment. This analysis provides insight into how different physical variables affect the model output and helps clarify the prediction mechanism of the proposed C-BL-R model. As shown in Fig 15, the input features are arranged along the vertical axis according to their overall importance, while the horizontal axis represents the SHAP value, indicating the contribution direction and magnitude of each feature to the prediction result. A positive SHAP value indicates that the corresponding feature increases the predicted capsizing moment, whereas a negative SHAP value indicates a decreasing effect. The color gradient from red to blue represents the feature value magnitude, with red indicating higher feature values and blue indicating lower feature values. Fig 15(a) illustrates the distribution and direction of feature contributions, while Fig 15(b) summarizes the overall feature importance based on mean absolute SHAP values.

The SHAP analysis provides further insight into the contribution of different input features to the prediction of capsizing moment. As shown in Fig 15(a), the historical capsizing moment variables *M*(t) and *M*(t-1) exhibit the largest SHAP values, indicating their dominant influence on the prediction. This observation is consistent with the temporal continuity of dynamic responses in the chamber-water-ship coupled system. For the excitation-related variables, *a*(t-1) shows a relatively wider SHAP distribution, indicating a nonlinear relationship between historical acceleration and the predicted capsizing moment. This behavior may be related to the dynamic response characteristics of the shiplift system, where transient excitation can influence the evolution of hydrodynamic loading through coupled structural and fluid responses.

In comparison, the geometric parameters, including L_2_/L_1_, B_2_/B_1_, H_2_/H, and H/H_1_, exhibit relatively smaller SHAP magnitudes but still provide additional information for distinguishing different chamber-water-ship configurations. As summarized in Fig 15(b), the SHAP results indicate that the proposed model is dominated by the autoregressive moment features *M*(t) and *M*(t − 1), while the acceleration, force, and geometric variables provide complementary information. Therefore, the present framework should be interpreted as a multivariate one-step autoregressive predictor rather than a long-horizon standalone forecasting model.

## Conclusions

This study addresses the challenge of efficiently predicting the capsizing moment of shiplifts under chamber-water-ship coupled interactions. It proposes a prediction method based on C-BL-R and systematically systematically evaluates its performance through numerical simulations and engineering case studies. The main conclusions are as follows:

1. A three-dimensional CFD model of the chamber-water-ship coupled system was established and validated against smoothed particle hydrodynamics simulations, with a maximum deviation of 4.1% in peak capsizing moment. Under El-Centro excitation, the peak capsizing moments predicted by the 3D model were markedly higher than those obtained from the traditional 2D simplified model for both the 3000 t light-load and 1350 t full-load conditions, indicating that two-dimensional simplifications may underestimate the capsizing moment under certain operating conditions. These results highlight the importance of three-dimensional analysis for reliable shiplift safety assessment.
2. Based on the numerically generated CFD dataset, a hybrid surrogate model integrating convolutional neural networks, bidirectional long short-term memory networks, and random forests was developed for one-step-ahead capsizing-moment prediction. Among eight candidate models, the proposed model achieved the best overall predictive performance on the test set, with a mean absolute error of 0.0761 ± 0.0024, a mean absolute percentage error of 1.110 ± 0.336%, a mean squared error of 0.0201 ± 0.0038, a root mean squared error of 0.1408 ± 0.0128, and a coefficient of determination of 0.9466 ± 0.0065.
3. Ablation studies showed that the C-BL-R model achieved improved predictive performance when using a 13 × 13 neuron configuration in combination with multi-window isolation forest preprocessing and Mapping Mountain Gazelle Optimizer-based hyperparameter tuning. In addition, the SHAP analysis showed that the historical capsizing-moment variables *M*(t) and *M*(t − 1) dominated the prediction, while *a*(t − 1) and the remaining acceleration, force, and geometric variables provided smaller complementary contributions.
4. Additional engineering cases showed that the proposed model captured the major variation trends and peak responses of capsizing moments, with peak prediction deviations below 4.8%. However, larger deviations were observed under ship-absence conditions, indicating that the current model is mainly applicable within the ship-present operating range. Additional ship-absence samples and broader operating conditions are required to further improve model robustness.

Overall, this study demonstrates the feasibility of CFD-informed, state-conditioned one-step-ahead capsizing-moment prediction. The current validation uses CFD-derived historical states as proxies for the measurements or state estimates required during practical operation. Therefore, field application will require an integrated real-time monitoring or state-estimation system and further experimental validation of measurement noise, synchronization, and computational latency. A principal limitation of the present study is the absence of physical-model or full-scale capsizing-moment measurements for the 19 shiplift configurations. Consequently, the current surrogate model should be regarded as an emulator of the CFD-generated response database. Future work should include scaled physical experiments, hydraulic-flume measurements, or field monitoring to quantify model-form uncertainty and assess the absolute accuracy of both the CFD framework and the surrogate model. In addition, future work should apply multiple seismic records to identical shiplift configurations over a controlled series of PGA levels, including additional rare-earthquake cases at and above 0.2g, to establish a statistically supported PGA applicability boundary and evaluate code-oriented engineering use.

## Supporting information

S1 Table Summary of the optimization algorithms used in this study. (XLSX)
